# Fundamentals, Synthetic Strategies and Applications of Non-Covalently Imprinted Polymers

**DOI:** 10.3390/molecules29153555

**Published:** 2024-07-28

**Authors:** Dongfeng Hong, Changzhao Wang, Liujing Gao, Caijian Nie

**Affiliations:** School of Food and Drug, Luoyang Normal University, Luoyang 471934, China; changzhaow@163.com (C.W.); 19937986200@163.com (L.G.); niecaijian@163.com (C.N.)

**Keywords:** molecularly imprinted polymers, non-covalent imprinting, synthetic strategies, applications

## Abstract

Molecular imprinting has emerged as an important and practical technology to create economical and stable synthetic mimics of antibodies and enzymes. It has already found a variety of important applications, such as affinity separation, chemical/biological sensing, disease diagnostics, proteomics, bioimaging, controlled drug release, and catalysis. In the past decade, significant breakthroughs have been made in non-covalently imprinted polymers, from their synthesis through to their applications. In terms of synthesis, quite a few versatile and facile imprinting approaches for preparing MIPs have been invented, which have effectively solved some key issues in molecular imprinting. Additionally, important applications in several areas, such as sensors, proteomics and bioimaging, have been well demonstrated. In this review, we critically and comprehensively survey key recent advances made in the preparation of non-covalently imprinted polymers and their important applications. We focus on the state-of-art of this technology from three different perspectives: fundamentals, synthetic strategies, and applications. We first provide a fundamental basis for molecular imprinting technologies that have been developed, which is extremely helpful for establishing a sound understanding of the challenges in molecular imprinting. Then, we discuss in particular the major breakthroughs within the last ten years (2014–2024), with emphasis on new imprinting approaches, what strengths the breakthroughs can provide, and which new applications the properties of the prepared non-covalently imprinted polymers are fit for.

## 1. Introduction

Molecularly imprinted polymers (MIPs) [1,2,3,4,5], which are chemical receptors synthesized in the presence of a template, have developed into important functional materials with antibody-like binding properties or enzyme-like catalytic activities and have found a wide range of applications from separation to sensing to proteomics. Due to the presence of the structure of nanoscale imprinted cavities that are highly complementary to template molecules, molecularly imprinted materials can provide excellent specificity and high affinity toward template molecules.

The development of molecular imprinting can be dated back to 1931, when Polyakov [6] reported that silica particles prepared from sodium silicate in the presence of organic solvent additives (benzene, toluene, or xylene) provided preferential binding affinity for the associated additive over the other two structural analogues. This is the earliest example of the concept of molecular imprinting found in the literature. At that time, Pauling was also pondering the origin of the selectivity of antibodies. Pauling [7] proposed a theory that antigens template the formation of antibodies and create structures complementary to themselves. By 1942, Pauling and co-workers [8] had prepared artificial antibodies using methyl blue dye as the antigen, which is the earliest known report of the connection between the biological templating that happens in nature and a synthetic imitation of this process. In 1949, inspired by Pauling, Dickey [9] reported silica gels prepared in the presence of dyes. After removing the patterning dye, the silica would rebind the same dye in preference to the others. Within the next 20 years, silica-based molecular imprinting continued, but the number of publications in this area remained low [10,11,12,13]. Clearly, all these efforts focused only on the sol–gel imprinting of silica. The main disadvantage of this method, using silica as a matrix, stems from the fact that, in principle, only two types of interactions are present: hydrophobic interaction with the siloxane groups, and hydrophilic interaction with the silanol groups. In all cases, the backbone is the same. In addition, the gel phase of silica in the template needed to be dried, which would cause shrinkage. 

To this end, research in molecular imprinting shifted away from silica in the 1970s, and the first report on molecular imprinting in organic polymers appeared in 1972 [14]. In 1981, Mosbach and co-workers [15] achieved a significant milestone by creating a molecularly imprinted organic polymer solely based on non-covalent interactions. This marked the second significant advancement in organic polymer imprinting, contrasting with the covalent approach previously employed by Wulff and his team. The introduction of this non-covalent imprinting technique sparked rapid growth in the field of molecular imprinting over the next few decades due to the wide availability of functional monomers and easy operation as compared with covalent imprinting. It is commonly held that both methods have their advantages and disadvantages. In 1995, Whitcombe and colleagues [16] introduced an intermediary technique that seemed to merge the benefits of both approaches. This method depends on covalent interactions in the polymerization phase, but utilizes non-covalent interactions during rebinding, and is known as semi-covalent imprinting. However, the non-covalent approach is still by far the most widely used approach in the synthesis of MIPs.

Since 2014, a number of significant breakthroughs in molecular imprinting have been achieved. Particularly, quite a few versatile and facile imprinting approaches for developing efficient MIPs have been invented, such as bead-forming imprinting, surface imprinting, accessible fragment imprinting, and so on, which has effectively solved bottle-neck issues in molecular imprinting and thereby allowed for the facile production of MIPs with highly desirable properties that were not well demonstrated before. These breakthroughs have not only largely enriched the approaches available for molecular imprinting, but have also significantly changed and developed the understanding and knowledge surrounding molecular imprinting. New MIPs have enabled important applications, such as disease diagnosis, proteomics and bio-imaging. Although quite a few reviews have been published in journals and books regarding different aspects of molecular imprinting, and several excellent reviews have been published on relevant topics [17,18,19,20,21,22,23], an authoritative, critical, and comprehensive review that surveys the major advances in recent 10 years and compares the strategies used in the newly invented approaches and the already existing approaches is apparently lacking. 

In this review, we focus on three different aspects of the state-of-art of this technology: fundamentals, synthesis, and applications. We first provide a fundamental basis of molecular imprinting, which has been used since the early beginnings of molecular imprinting and is extremely helpful for a sound understanding of the challenges in molecular imprinting and how the current breakthroughs have solved these challenges. Then, we describe the major breakthroughs since 2014, with emphasis on new imprinting approaches and the important applications of molecularly imprinted materials. Some representative references that describe better fundamentals, synthesis methods, and applications were selected for use this work.

## 2. Fundamentals of Molecular Imprinting

In general, molecular imprinting is a process where functional monomers and cross-linkers are co-polymerized in the presence of a template (the imprint molecule). The functional monomers initially form a complex with the imprint molecule. After polymerization, functional groups are held in position by the highly cross-linked polymeric structure. After the imprinted template molecules in the MIP are removed, cavities complementary to the molecular shape and functionalities of the template are left behind in the polymer. As such, a molecular memory is introduced into the polymer, which is thereafter capable of rebinding the template with high specificity. With the development of molecular imprinting technology over the decades, a series of fundamentals issues relating to molecular imprinting have been settled and clarified. This section mainly describes the four aspects of the fundamental basis of molecular imprinting, including imprinting type (classified in terms of ligand–template interactions), reagents for non-covalent imprinting, imprinting strategies, and binding properties. These general aspects are essential for sound understanding of the synthesis, properties, and applications of molecularly imprinted materials.

### 2.1. Reagents for Non-Covalent Imprinting

The non-covalent imprinting approach utilizes only non-covalent interactions, such as hydrogen bonds, ionic interactions, hydrophobic interactions, and van der Waals forces, to generate adducts of functional monomers and templates in solution in the molecular imprinting process. This approach is quite flexible due to its broad selection of functional monomers. Another advantage is its simplicity in operation, owing to the absence of complicated synthetic chemistry between functional monomers and templates. Molecularly imprinted polymers (MIPs) created through non-covalent imprinting are typically expected to exhibit faster rebinding kinetics compared to those produced through the covalent method. However, one drawback of non-covalent imprinting is the potential for heterogeneity in binding sites due to the equilibrium system present in the pre-polymerization complex.

**Figure 1:**
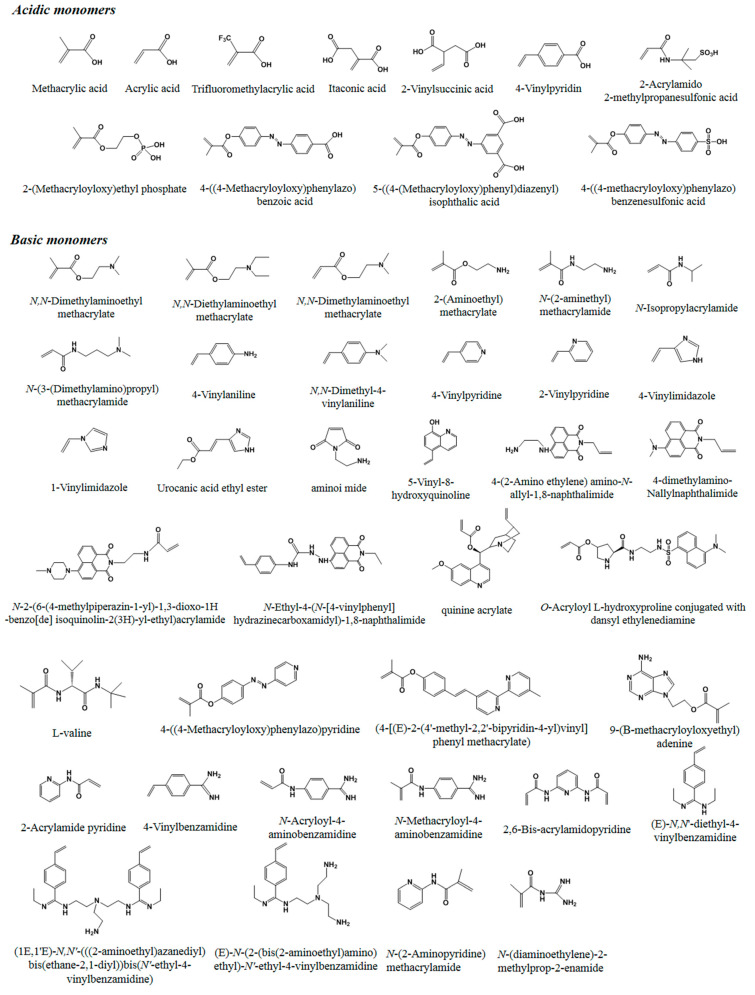

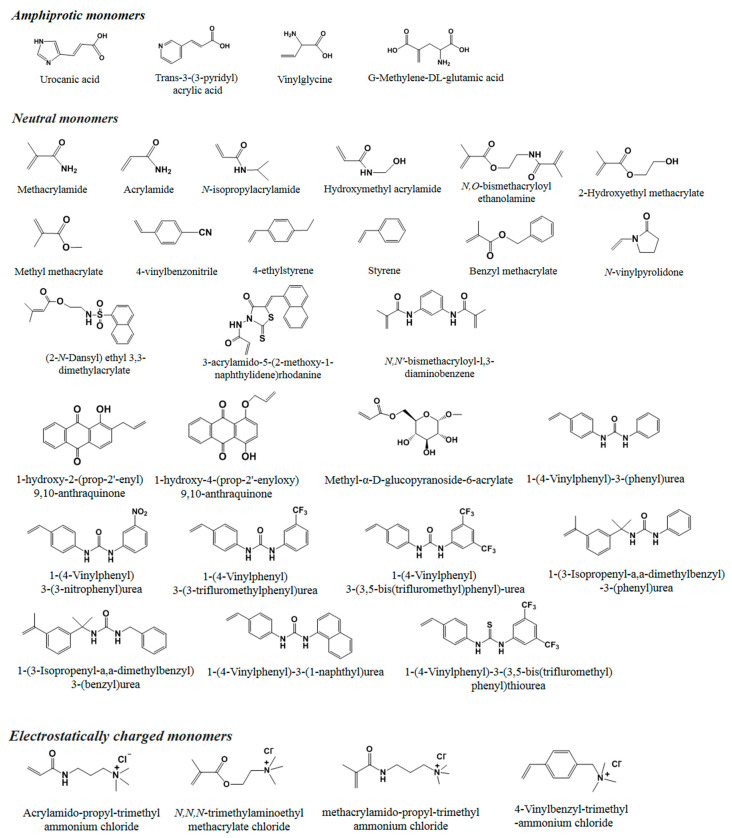
Molecular structures of functional monomers containing a vinyl double bond employed in non-covalent molecular imprinting.

Generally, the used reagents for non-covalent imprinting include functional monomers, templates, cross-linkers, and initiators. Up until now, many functional monomers have been used in non-covalent imprinting. Generally, a functional monomer consists of two types of essential moieties: one is a recognition moiety, such as a carboxyl, amino, or carbamide group, and the other is a reactive moiety, such as a vinyl double bond, silicon hydroxyl, or mercapto group. Functional monomers can be classified into five categories, according to the nature of the recognition moiety: (1) acidic, (2) basic, (3) amphiprotic, (4) neutral, and (5) electrostatically charged. Functional monomers with a vinyl double bond are the most widely used. Among them, the most commonly used monomers for non-covalent imprinting mainly include acidic methacrylic acid (MAA), acrylic acid (AA), neutral methacrylamide (MAAm) and acrylamide (AAm), due to their unique characteristics, ability to act as a hydrogen-bond donor and acceptor, and low cost. In order to improve the specificity of MIPs for some specific templates, many other monomers, capable of forming strong interactions with templates, have been reported [24,25,26,27,28,29,30,31,32,33,34,35,36,37,38,39,40,41,42,43,44,45,46,47,48,49,50,51,52,53,54,55]. Figure 1 shows the structures of a variety of functional monomers with a vinyl double bond that have been used in non-covalent imprinting.

It should be pointed out that dicarboxylic acid-based monomers could provide a higher binding force with templates due to the presence of stronger hydrogen bonding and ionic interactions compared with single carboxylic acid-based monomers [56]. In addition to carboxylic acid-based monomers, some more acidic monomers, such as sulphonates, phosphonates, phosphates, etc., have also been used to prepare MIPs, which are particularly advantageous for positively charged templates, because these could be particularly used to form ion pairs in imprinting under aqueous conditions [57,58,59]. Additionally, carboxylic acid-based photoresponsive monomers, such as 4-((4-methacryloyloxy)phenylazo)benzoic acid (MPABA) [60] and 5-[(4-(methacryloyloxy)phenyl)diazenyl]isophthalic acid (MAPDIA) [61], and sulfonic acid-based monomers, such as 4-MAPASA, could be utilized for the development of photoresponsive MIPs.

Among these basic monomers, *N*-ethyl-4-(*N*-[4-vinylphenyl] hydrazinecarboxamidyl)-1,8-naphthalimide is particularly advantageous for carboxylate-containing templates due to the presence of a urea binding site in the monomer. Furthermore, a variety of chiral monomers derived from L-valine have been employed in the synthesis of MIPs for separating dipeptide diastereomers. In such instances, the inherent configurational chirality present in the side chains of the polymer enables them to act as chiral selectors, with imprinting serving to amplify selectivity. In addition, basic form-based azobenzene (azo)-containing monomers such as 4-((4-methacryloyloxy)phenylazo)pyridine (MAzoPy) could be used to prepare photoresponsive MIPs [24]. Amidines or guanidines are another type of monomer with a unique structure, and have been developed for highly efficient imprinting use because they are able to form multiple hydrogen bonds with templates [25,26,27,28,29,30,31].

**Figure 2:**
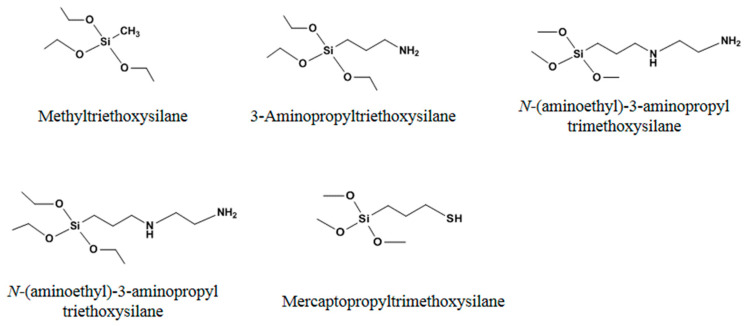
Functional monomer architectures employed in the sol–gel process for non-covalent molecular imprinting.

In addition to free radical polymerization in molecular imprinting, the sol–gel reaction with silicon hydroxyl has also often been used to develop effective MIPs. The most commonly used monomers to date are listed in Figure 2.

In addition to non-covalent imprinting with a single functional monomer, imprinting with combinations of monomers has been widely used in order to improve affinity and selectivity. It is highly attractive to combine the specific interaction potential of a variety of different monomers. In one study, the combinational monomers of the weakly basic 2-vinylpyridine and the acidic and hydrogen-bonding MAA were used to develop better MIPs for amino acid derivatives as compared with either monomer alone [40]. Combinations of AAm and 2-vinylpyridine have also been used in the effective imprinting of amino acid derivatives [41].

**Figure 3:**
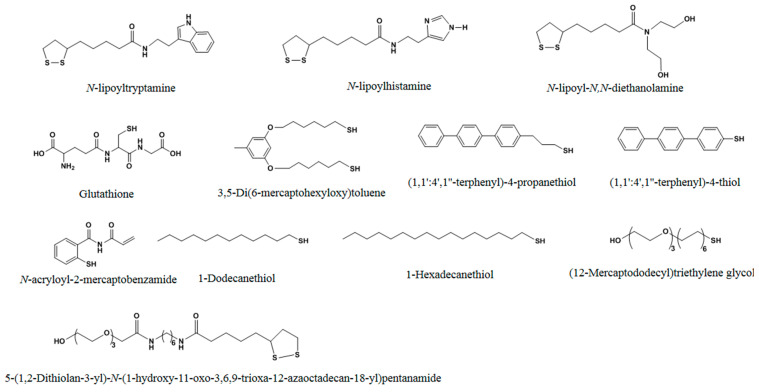
Structures of functional monomers with mercapto groups used in non-covalent molecular imprinting.

Functional monomers with mercapto group (Figure 3) also have been used to prepare MIPs through self-assembly in a unique way. These monomers form self-assembled monolayers (SAMs) rather than undergoing polymerization. Compared to the polymerization method, this approach can offer a higher level of uniformity in binding sites and more effective quantitative removal of templates.

**Figure 4:**
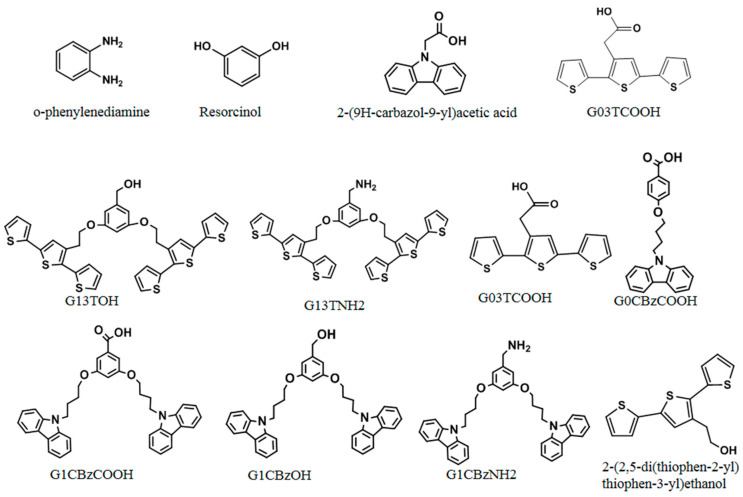
Structures of the functional monomers used in electropolymerization in non-covalent molecular imprinting.

In addition, MIPs can be prepared by electropolymerization using reactive moiety. Generally, cross-linkers are not needed when using this method. To date, a series of electropolymerizable terthiophene (e.g., 2-(2,5-bis(thiophen-2-yl)thiophen-3-yl)ethanol, G03TCOOH, G13TOH, and G13TNH2) and carbazole monomers (e.g., 2-(9H-carbazol-9-yl) acetic acid, G0CBzCOOH, G1CBzCOOH, G1CBzOH, and G1CBzNH_2_) have been applied in the imprinting of template molecules [45,46,47], as listed in Figure 4. 

**Figure 5:**
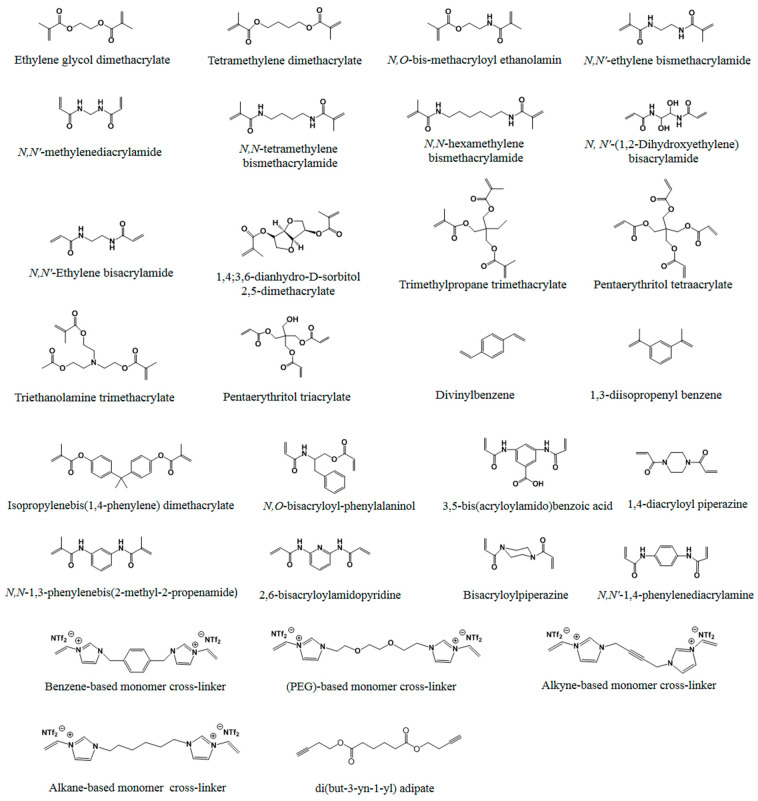
Structures of cross-linkers used in free radical polymerization in non-covalent molecular imprinting.

**Figure 6:**
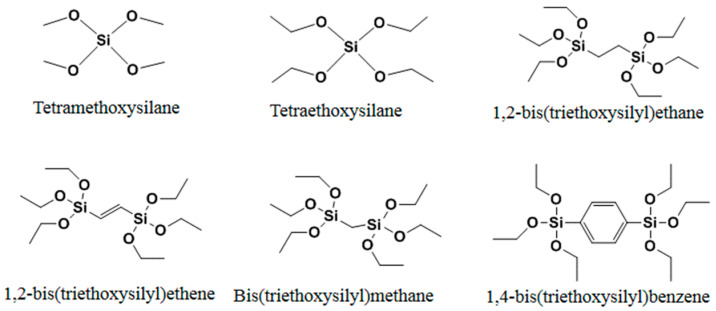
Structures of cross-linkers used in sol–gel process in non-covalent molecular imprinting.

In the process of polymerization, a cross-linker is used to fix functional monomers around template molecules, forming a highly cross-linked rigid polymer that remains even after the removal of templates. The type and the amount of cross-linker used have a great effect on the selectivity and binding capacity of MIPs [48,49]. Usually, an amount of cross-linker that is too low will result in unstable mechanical properties due to the low cross-linking degree, while an extremely high amount of cross-linker will reduce the binding capacity of MIPs. Thus, the type and amount of cross-linker employed need to be carefully optimized. So far, the mostly commonly used cross-linkers in free radical polymerization are shown in Figure 5. In addition to the above mentioned cross-linkers in free radical polymerization, those in sol–gel processes could also be used to prepare MIPs. Tetraalkoxysilane monomers of the form Si(OR)4, or silsesquioxane precursors of the form (RO)_3_SiR’ Si(OR)_3_, can cross-link with functional monomers to create the matrix shown in Figure 6. The most widely used cross-linkers includes TMOS and TEOS.

**Figure 7:**
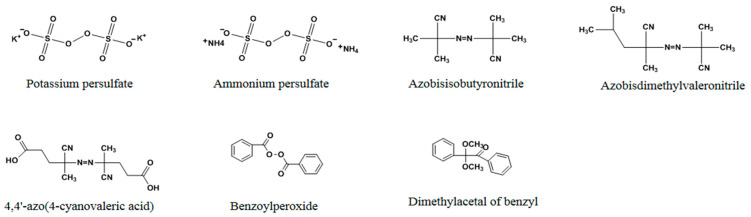
Structures of initiators frequently used in molecular imprinting.

In non-covalent imprinting, initiators are required for free radical polymerization. Initiators function either thermally or photochemically. In addition to peroxy compounds (ammonium persulfate, potassium persulfate), azo compounds, such as azobisisobutyronitrile (AIBN), azobisdimethylvaleronitrile (ADVN), and 4,4′-azo(4-cyanovaleric acid) (ACID) [48], are extensively used as initiators (Figure 7). Among them, AIBN is the most widely used initiator.

### 2.2. Imprinting Strategies

Currently, the most widely used strategies for molecular imprinting can be classified into three major types: bulk imprinting, bead-forming imprinting, and conventional surface imprinting. 

#### 2.2.1. Bulk Imprinting

In many molecular imprinting scenarios, the template, functional monomers, and the cross-linker are mixed together, and the mixture is polymerized into a polymer. Then, the polymers obtained by bulk polymerization are crushed, ground, and sieved to an appropriate size. Such an imprinting methodology is called bulk imprinting, and is also known as the “one-pot” method. Bulk polymerization was the most widely used approach in the early stages of molecular imprinting, and it is still widely used today, because it is relatively straightforward and easy to handle technically. Bulk polymerization has proven to be an important preparation technique for MIPs for a large number of small templates, including for drugs such as penicillin G [62] and ibuprofen [63], herbicides such as phenoxy acetic acids [64] and atrazine [65], flavonoids such as quercetin [66,67], and even various mycotoxins, such as zearalenone [68,69] and moniliformin [70]. However, bulk imprinting is associated with several apparent drawbacks. Although the initial preparation is simple, the subsequent processing of bulk polymers is time consuming, as the resulting monolithic structure has to be ground and sieved into particles of suitable sizes. The grinding of bulk polymers also leads to a wide particle size distribution and irregular particle shapes, which may be unfavorable for some applications, such as chromatographic separation [71]. In addition, bulk polymerization is only applicable on a small laboratory scale. For mass production of MIPs, scalable preparation methods are needed. Moreover, bulk imprinting suffers from incomplete template removal, a small binding capacity, and slow mass transfer. These drawbacks apparently hinder the widespread application of bulk imprinting. In chromatographic analysis, the in situ preparation of monolithic MIPs has been introduced to overcome some of the drawbacks of traditional bulk MIP production. This method offers the opportunity of fabricating MIP-based stationary phases for liquid chromatographic or electrochromatographic separations without the grinding, sieving, and packing of particulate material into a column or capillary. To achieve high template removal and maintain sufficient liquid flow through the column, the in situ prepared MIPs should have a macroporous structure [72,73,74,75]. 

#### 2.2.2. Surface Imprinting

As mentioned above, the obtained beads using bulk imprinting and bead-forming imprinting can result in incomplete template removal, a small binding capacity, and slow mass transfer. Particularly, the imprinting of water-soluble biomacromolecules, such as proteins, using these two strategies is rather ineffective. In order to address these issues, surface initiated imprinting can be utilized, where the template molecules are located on or near the material’s surface. The choice of supporting material plays a crucial role in surface-initiated imprinting. Compared to traditional MIPs, surface-imprinted polymers possess not only a higher binding capacity, but also faster mass transfer and binding kinetics. Many material formats have been used as supports in the surface-initiated imprinting process, such as polymer beads [76], monoliths [77], silica nanoparticles [78,79], Fe_3_O_4_ magnetic nanoparticles [80,81,82], quantum dots (QDs) [83,84], glass membranes [85,86,87,88], and microplates [89,90].

### 2.3. Binding Properties

Adsorption isotherms are widely used to describe the binding behavior of molecularly imprinted materials. Based on adsorption isotherms, important parameters, such as imprinting factor, binding capacity, and binding affinity, can be calculated. In addition, other parameters, including cross-reactivity, imprinting efficiency, template usage efficiency, and binding kinetics, are also used in the assessment of the performance of molecularly imprinted materials. Here, we describe six important aspects involved in the property characterization of MIPs. In the literature, many papers have reported only a small portion of these necessary property characterizations, which makes it very difficult for readers to make a sound and comprehensive comparison among different molecularly imprinted materials. Thus, we appeal that all researchers publishing studies on molecular imprinting should provide all of the binding parameters as often as possible. 

#### 2.3.1. Binding Capacity and Imprinting Factor 

**Figure 8:**
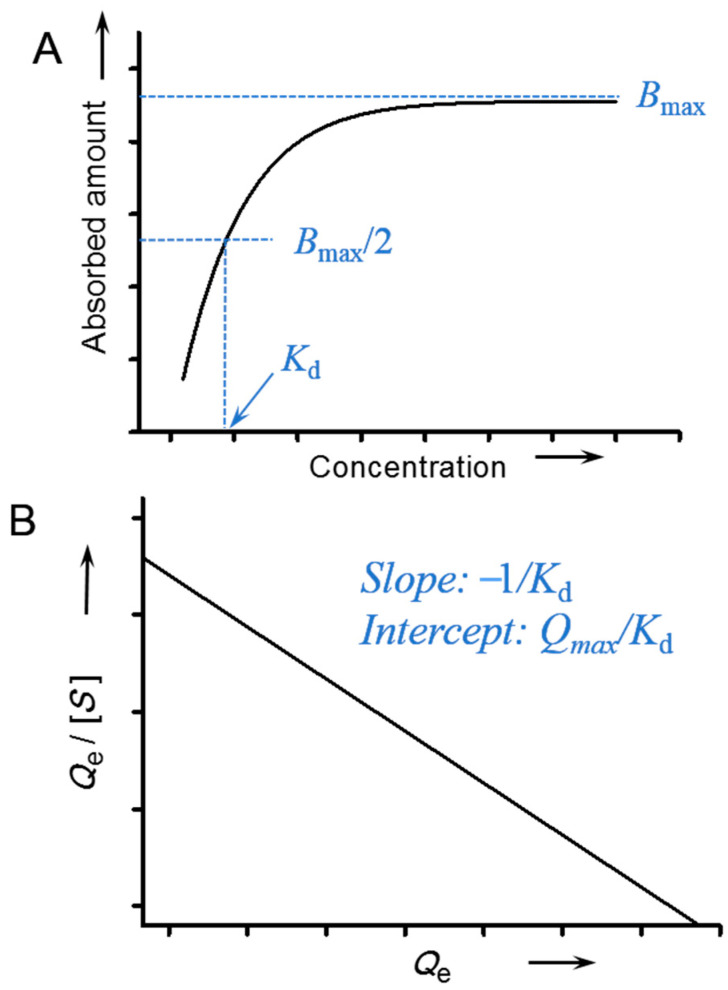
Schematic illustrations of (**A**) adsorption isotherms and (**B**) Scatchard analysis.

Binding capacity (*B_max_*) is the maximum amount of analytes that a MIP can adsorb under the saturated condition, which can be obtained directly from an adsorption isotherm (Figure 8A). 

The imprinting factor reveals the difference between the binding capacities of imprinted and non-imprinted materials. The higher the imprinting factor is, the more effective the imprinting approach is. The imprinting factor is calculated as the ratio of the *B*_max_ for an imprinted material versus that for a non-imprinted material.

#### 2.3.2. Binding Affinity 

Binding affinity, or binding strength, is the most important parameter of molecularly imprinted materials. A high binding affinity implies that the MIP can capture the target at a very low concentration, which is greatly beneficial for practical applications. Binding affinity is usually described using the disassociation constant (*K_d_*). If a binding isotherm has been established, the *K_d_* can be calculated through appropriate mathematical fitting or empirical estimation. The most commonly used mathematical fitting for *K_d_* determination is the Scatchard analysis. The Scatchard relationship can be established using the following equation:

(1) $$\frac{Q_{e}}{\left[S\right]}=\frac{Q_{max}-Q_{e}}{K_{d}}$$

In this equation, *Q_e_* represents the amount of analyte bound to the imprinting material; [*S*] is the free concentration of analyte at adsorption equilibrium; *Q_max_* is the theoretical saturated adsorption capacity (*Q_max_*); and *K_d_* is the dissociation constant. By plotting *Q_e_*/[*S*] versus *Q_e_*, the values of *K_d_* and *Q_max_* can be determined from the slope and intercept, respectively (Figure 8B).

Sometimes the binding data do not obey a straight line in the Scatchard plot. In these cases, alternative fitting equations, such as the Hill equation and the logistic equation, can be used for *K_d_* determination. The Hill equation is given below:

(2) $$y=\frac{B_{max}x^{n}}{x^{n}+{K_{d}}^{n}}$$

where *B_max_* is the maximum specific binding, and *n* is the Hill slope. The logistic equation is given below:

(3) $$y=\frac{a-d}{1+{\left(\frac{x}{c}\right)}^{b}}+d$$

where *y* is the signal intensity; *x* is the concentration; and *a*, *b*, *c*, and *d* are the signal intensity when *x* = 0, the “slope factor” that determines the steepness of curve, and the concentration halfway between *a* and *d*, which gives the *K_d_* value and the signal intensity for an “infinite” concentration, respectively. The empirical method is also often employed for rough estimation of *K_d_*; this is calculated using the free concentration at the *x* axis that gives half of the value of the *B_max_* (Figure 8A).

In addition to the above data fitting methods, instrumental methods, including QCM and surface plasmon resonance (SPR), have been widely used for the *K_d_* measurement. Bio-layer interferometry (BLI) has also been recently demonstrated to be an efficient approach for the measurement of binding strength. It should be noted that all these methods require that the MIP to be evaluated has been prepared in a thin layer form, and thereby they are not applicable to other material forms.

#### 2.3.3. Cross-Reactivity

In immunology, cross-reactivity is one of the most critical aspects of antibodies and specifically represents the reaction between an antibody and an antigen, which differs from the immunogen. For molecularly imprinted materials, cross-reactivity is also an important parameter for the validation of imprinted materials. Cross-reactivity is normally determined by comparing the binding response to a range of similar analytes and is expressed as a percentage. In practice, the calibration curves of a selection of related compounds should be produced for quantified comparison. The results provide an estimate of the response of the material to possible interfering compounds. For most of the reported MIPs, the cross-reactivities are around 10–40%. Therefore, cross-reactivity of less than 10% can be considered as representative of excellent specificity.

#### 2.3.4. Imprinting Efficiency and Template Usage Efficiency 

Imprinting efficiency and template usage efficiency are two important criteria for the evaluation of a molecular imprinting approach. Imprinting efficiency is defined as the ratio of the number of imprinted cavities of the molecularly imprinted material to the total number of template molecules used in the synthetic procedure. Imprinting efficiency reflects the portion of well-formed binding cavities among all used template molecules. The reported efficiencies of different imprinting approaches vary from a few percent to almost one hundred percent. However, imprinting approaches with ultra-high efficiency are often associated with sophisticated and strict synthetic procedures. Thus, imprinting approaches with ultra-high efficiency are not always the best choice. However, for particularly rare and expensive imprinting templates, these approaches with ultra-high efficiency are of course highly favorable.

Template usage efficiency is defined as the percentage of the amount of recovered template compared to the amount of template used in the synthetic procedure. The template usage efficiency indicates the recovery of the template. Unfortunately, only a few references have reported on this parameter. Similar to ultra-high efficiency imprinting approaches, certain delicate imprinting approaches are necessary to achieve high template usage efficiency. 

#### 2.3.5. Binding Kinetics

In a reversible reaction, the chemical kinetics describe how fast the reaction can reach its equilibrium, and are related to the on- and off-rates of the binding. Some instrumental methods, such as QCM, SPR, and BLI, can provide association rate constants (*k*_on_) and dissociation rate constants (*k*_off_). The relationship of these constants with the *K*_d_ value is described in the following equation. However, in most publications on MIPs, these parameters have not been provided, making comparison between different MIPs impossible.

(4) $$K_{d}=\frac{k_{\mathrm{off}}}{k_{\mathrm{on}}}$$

Fast binding kinetics usually greatly benefit MIP-based fast analysis. For convenience, equilibrium times are often used for the assessment of the binding kinetics of MIPs. To obtain these measurements, a certain amount of an MIP to be evaluated is incubated with the saturated analyte solution for different durations, and then the on-rate of binding is determined by the minimum incubation time for equilibrium. For off-rate determination, the analyte-bound MIP is incubated with eluting solution for different time periods, and the off-rate of binding is established as the minimum time to remove all of the bound analytes. Up until now, most reported molecularly imprinted materials have been found to reach their equilibrium within several hours, which is comparable to most antibodies. However, in order to meet the requirements of fast analysis, new materials with fast binding kinetics are of great importance.

## 3. Synthesis Methods of Non-Covalently Imprinted Polymers

The abovementioned four imprinting strategies have been widely employed to prepare MIPs and have found many applications. However, there are particular applications for which they may not work well. In the past five years, a variety of new molecular imprinting approaches have emerged. Some of these new approaches are improved versions of the imprinting strategies discussed above. Some are combinations of these strategies, which inherit the merits of these strategies and overcome their deficiencies to some extent. In addition, some unique imprinting approaches have also been developed. With these new methods, MIPs in new material forms with more desirable properties have been prepared, which has enabled a variety of new applications.

### 3.1. Bead-Forming Imprinting

As mentioned above, the majority of traditional MIPs are in the format of irregular particulates obtained by grinding cross-linked polymer monoliths. In order to obtain regular, spherical particles with narrow size distributions, bead-forming imprinting has become an effective strategy. Bead-forming imprinting comprises three main approaches: precipitation [91,92,93,94], suspension [95,96,97], and emulsion [98,99,100]. In recent years, these approaches have undergone significant development.

Precipitation polymerization is a stabilizer-free polymerization process which starts from a homogeneous solution containing monomer, template, cross-linker, and initiator. The relatively straightforward method of precipitation polymerization has become an increasingly popular method for producing beaded MIPs. The omission of stabilizer greatly simplifies post-polymerization processing, since it does not require removing stabilizer from the bead surface. Another advantage is that the use of polar aprotic solvents in precipitation polymerization is capable of preserving non-covalent interactions between the template and monomer. These advantages have made this method a widely applicable imprinting approach. However, a disadvantage is the high dilution conditions required to avoid agglomeration of the growing particles. Recently, several uniformly sized imprinted beads for small molecules were prepared by thermal precipitation polymerization [101,102,103]. Results from binding experiments proved that beaded MIPs exhibited high specificity and affinity for templates. However, the uptake and release of template requires complex operation. To facilitate template uptake and release, Bakker and colleagues developed photoresponsive ion-imprinted polymer (PIIP) for the uptake of metal ions. In traditional precipitation polymerization, it is not easy to control the uniformity of beads in size and shape. Controlled/living radical polymerization (CRP), a type of unique precipitation polymerization, represents an advanced technique in the preparation of well-defined polymeric materials with desirable architectures. CRP has been used in molecular imprinting. Reversible addition–fragmentation chain-transfer (RAFT) and atom transfer radical polymerization (ATRP) are two types of CRP that possess wide applicability and the benefit of requiring mild reaction conditions [104]. In recent years, it has been considered that RAFT might be a more ideal candidate than ATRP, not only due to its versatility and simplicity, but also because its polymerization products are free from contamination with the metal catalysts used in ATRP. It has been found that beads with a uniform particle size can be obtained more easily using RAFT or ATRP polymerization as compared with traditional precipitation polymerization [105]. In recent years, RAFT and ATRP techniques have been widely utilized to synthesize narrowly dispersed MIP beads, owing to their intrinsic advantages over traditional precipitation polymerization [106,107,108,109,110]. Zhang and co-workers made a great breakthrough in the direct preparation of MIP beads [109,110]. To suppress the significant hydrophobicity driven by nonspecific interactions between the MIPs and template molecules, they grafted ultrathin hydrophilic poly(2-hydroxyethyl methacrylate) [107], poly(glyceryl monomethacrylate) [109], and poly (N-isopropylacrylamide (NIPAAm) [110] brushes onto the MIP microspheres via surface-initiated RAFT polymerization, which could exhibit specific binding in pure aqueous media. However, these water-compatible microspherical MIPs demand multi-step synthesis. To this end, Zhang and co-workers developed an efficient one-pot approach to synthesize water-compatible and narrowly dispersed MIP microspheres with surface-grafted hydrophilic polymer brushes by facile RAFT precipitation polymerization [111]. However, the template binding properties of these water-compatible MIPs could not be controlled under photoswitching conditions in aqueous media. To overcome such a limitation, Haupt and co-workers [112] proposed a new method of photoinitiated ATRP to prepare MIPs for the recognition of drugs such as testosterone and S-propranolol. The reaction was carried out at room temperature and was highly compatible with acidic monomers, which overcame the two major limitations of using ATRP with MIPs, effectively widening the range of functional monomers and molecular templates that can be used in ATRP.

**Figure 9:**
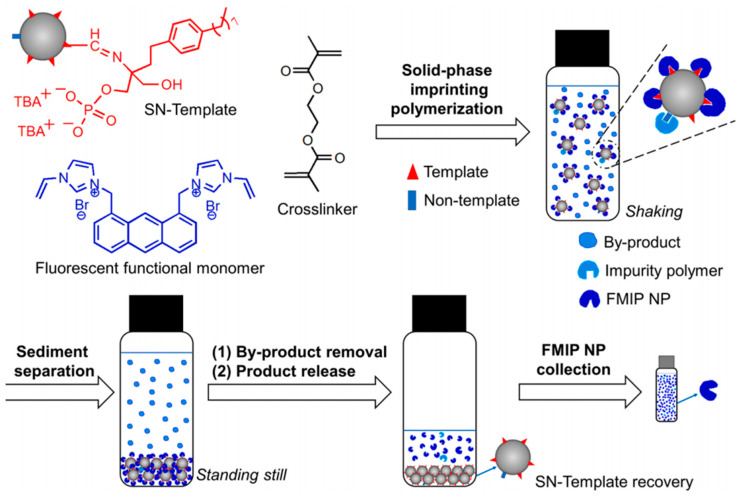
Schematic of a sedimentation-based solid-phase imprinting strategy for preparing fluorescent molecularly imprinted nanoparticles (FMIP NPs) targeting the phospholipid. SN, silica nanosphere; template, fingolimod phosphate salt; TBA, tetrabutylammonium. The non-template is the residual amino or aldehyde group (in low amounts), which might generate minor impurity polymer [113].

Conventional precipitation polymerization is usually performed in solution, which causes inevitable problems, including binding site heterogeneity, poor accessibility, and template residues. Sellergren and co-workers [113] developed a solid-phase imprinting strategy for preparation of molecularly imprinted polymer nanoparticles to solve these problems. Using silica nanospheres (SNs) as solid supporting material, template molecules (phospholipids) were covalently immobilized on the surface of the support (Figure 9). The polymerization process was based on the use of template-modified SNs that were dispersed in the reaction medium by non-shearing agitation. In the presence of fluorescent functional monomers with imidazolium groups, designed to interact with the immobilized template, growing polymer chains were attracted to the SNs, and after further monomer recruitment, finally resulted in surface-imprinted nanoparticles.

**Figure 10:**
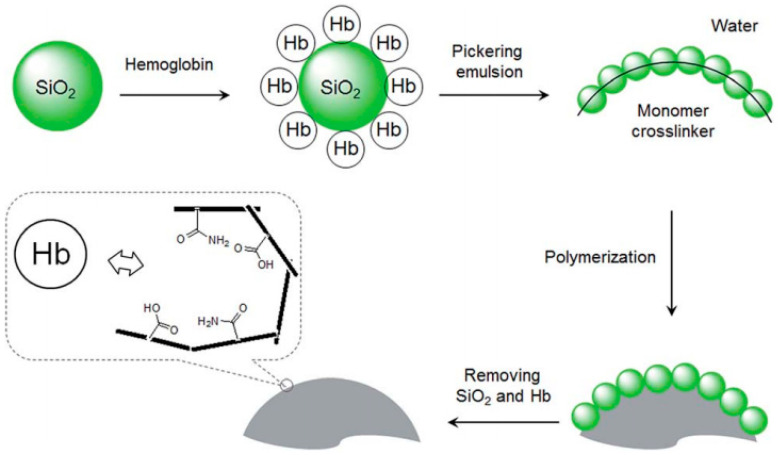
Schematic illustration of the interfacial protein imprinting process [119].

In addition to precipitation polymerization, emulsion polymerization and suspension polymerization are also effective methods for preparing narrowly dispersed hydrophilic MIP beads due to good control of particle sizes. Through emulsion polymerization, small MIP beads with a narrow size distribution can be generated. The use of water or oil as the continuous phase in emulsion polymerization requires that relatively strong interactions between the template and functional monomers are maintained during the process of bead formation. In conventional emulsion, discrete liquid droplets are stabilized by surfactants. BSA-imprinted microsphere [114] and pepsin-imprinted polymer beads [115] are prepared via emulsion polymerization stabilized by surfactants. However, the type of emulsion obtained using surfactants as stabilizers is not particularly stable. Pickering emulsions, a novel type of emulsion, can be classified as either oil-in-water (O/W) or water-in-oil (W/O), and are stabilized by solid particles. Compared to emulsions using small molecule surfactants, Pickering emulsions are often more stable and are thus simpler to employ in the creation of intricate micro and nanostructures. MIP microspheres selective for small organic molecules have been synthesized by Pickering emulsion polymerization [116,117,118]. In these works, SiO_2_ or TiO_2_ nanoparticles were used as stabilizers in forming a stable O/W emulsion. However, the template imprinted sites could not be completely situated on the surface of MIPs due to the presence of the weak non-covalent interaction between template and silica nanoparticles. In addition, the hydrophilic MIP beads had low template removal efficiency and low mechanical strength. To address these issues, Ye and co-workers subsequently developed a new method for the synthesis of protein-imprinted sites on bead surface by presenting the protein template on the surface of the stabilizing silica nanoparticles via oil-in-water (O/W) Pickering emulsion polymerization [119]. This new method involved the use of protein coated silica as the stabilizing particles to establish an O/W Pickering emulsion. The oil phase contained a cross-linking monomer and initiator, and the functional monomer that interacted with the protein template was enriched at the oil–water interface due to the adsorption of the protein template to the silica nanoparticles (Figure 10). After free radical polymerization of the oil phase, the protein-silica particles were removed to leave Hb-imprinted sites on the polymer beads surface. The imprinted polymer beads prepared via the Pickering emulsion polymerization exhibited protein-selective binding sites on their surface. Notably, biological particles, such as bacteria, have also been imprinted by Pickering emulsion polymerization [120]. Bacteria treated with acryloyl-functionalized chitosan have been used to stabilize an O/W emulsion in which cross-linking monomers copolymerized into dispersed beads while in the O/W interface due to the presence of template bacteria. Imprinted cavities complementary to the template were formed for recognition of the biological particles. However, the process of blending polymer and emulsion nanoparticles is intricate, involving steps such as emulsion breaking, particle washing, and product drying. Mixing emulsion nanoparticles directly with polymer can prove challenging due to the difficulty in eliminating surface-bound impurities, often resulting in particle coagulation and deformation.

On the other hand, suspension polymerization has the capability to address the aforementioned issues. Suspension polymerization can also be used to prepare MIP beads with a narrow size distribution due to the advantage of the simple control over reaction conditions. Generally, compared with emulsion polymerization, suspension polymerization gives larger beads and a broader distribution in particle size. Recently, many novel MIP microspheres for small molecules have been developed by suspension polymerization [121,122,123]. He and co-workers developed fragment-imprinted microspheres of 2,6-dichloropyrimidine as templates for the determination of sulfonamides in milk samples by suspension polymerization [124]. The MIP microspheres exhibited high group selectivity toward sulfonamides containing 2,6-dichloropyrimidine. In addition to a substructure of the target sulfonamides, a sulfonamide analog, such as 4-sulfa-6-chloropyrimidine, was also used as a template to prepare MIP microspheres for sulfonamides by suspension polymerization [125]. However, the MIPs prepared using the target substructure or target analog as a template exhibited low binding capacity and low binding affinity. To address these challenges, a novel approach was developed involving the synthesis of a new ionic liquid-modified target for analog-imprinted microspheres through aqueous suspension polymerization. This utilized phenylephrine as a dummy template and the ionic liquid 1-allyl-3-ethylimidazolium bromide as a co-functional monomer. The resulting MIP beads showed enhanced affinity and binding capacity for clorprenaline and clenbuterol, attributed to the ionic liquid’s characteristics of low volatility, high stability, and good solubility for organic and inorganic compounds.

Additionally, Zhao and co-workers proposed a unique bead-forming imprinting strategy based on surface-cross-linked micelles [126]. Different from conventional micelles, these micelles consisted of a self-synthesized surfactant possessing multiple functions. Firstly, the hydrophilic head had three alkyne groups, which could form micelles in water with a high density of alkyne on the surface for cross-linking and adducting hydrophilic ligands. Secondly, the hydrophobic chain of photopolymerization served as the monomer, providing an active group to interact with the template. Surface cross-linking and sugar derivatives functionalization were easily performed through click-reaction with a diazide regent/group as the cross-linker and hydrophilic ligands. After being subjected to UV irradiation, the micelles copolymerized into nanoparticles. The nanoparticles were finally precipitated in acetone, followed by template removal in methanol/acetic acid solution. In this strategy, with the mixture of a cationic cross-linkable surfactant and an anionic template, their electrostatic interactions made it easy to incorporate the template inside the micelle and ultimately inside the MIP, while the hydrophobic interaction oriented the hydrophobic part of the template within the hydrophobic core of the micelle. Both factors improved the affinity of the imprinted pocket towards the template. The nanoparticles, in stoichiometry, contained one binding site per particle and displayed highly selective binding among structural analogues [127]. Owing to the hydrophilic ligands surrounding the micelle core, the resulting materials exhibited excellent water solubility, facilitating their use as protein-like receptors for the recognition of targets in water. Moreover, because the radius of the MIP was within the Förster distance of many fluorophore pairs, a MIP modified with an fluorophore was developed to detect analytes through Förster resonance energy transfer (FRET).

### 3.2. Solid-Phase Synthesis

**Figure 11:**
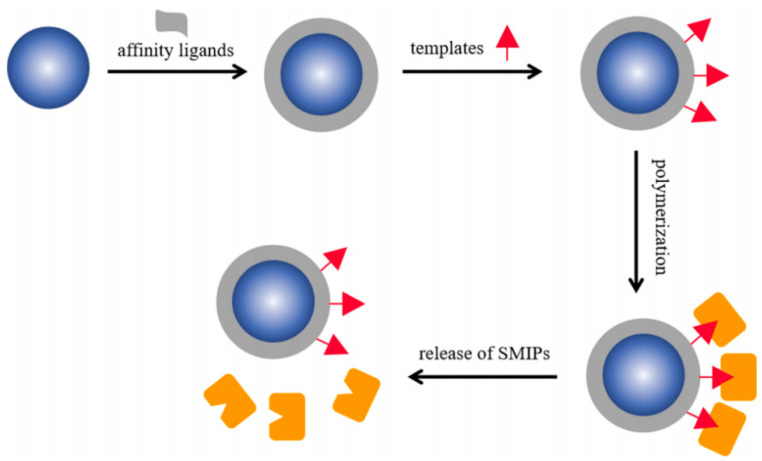
Schematic representation of solid-phase synthesis SMIPs.

Traditional MIPs obtained by bulk imprinting have some defects, including low adsorption capacity, poor site accessibility, restricted mass transfer, and irregular morphology, which limit their development. Surface MIPs (SMIPs) possess recognition cavities situated on or close to the surface of substrate materials, and have received wide attention [128,129]. Compared to bulk imprinting, SMIPs obtained by surface imprinting result in the complete removal of templates and provide excellent access to target molecules. Thus, SMIPs have many beneficial features, including a large surface area, the capacity for fast mass transfer, and high adsorption capacity and efficiency [130]. Solid phase synthesis processes for SMIPs are the most widely used synthetic strategies, and are described in Figure 11. Initially, the templates are anchored to a solid substrate in a solution. Then, these templates are placed in contact with a polymerization system (e.g., monomers, initiators, etc.), and polymerization is carried out under suitable conditions (e.g., heat and UV irradiation). Finally, these surface-imprinting materials remain bound to the solid substrate until an affinity-based purification of the SMIPs is performed. The benefits of the proposed solid-phase synthesis approach include its versatility, ease of automation and standardization, uniform binding properties, elimination of potential template-related contamination of the product, and the possibility of template re-use. Thus, these SMIPs can be applied in different areas, such as for imprinted biomacromolecules. As an example, dual-oriented solid-phase molecularly imprinted nanoparticles have been successfully used as an artificial receptor for recognition of adenosine monophosphate [131].

### 3.3. Target Substructure Imprinting

**Figure 12:**
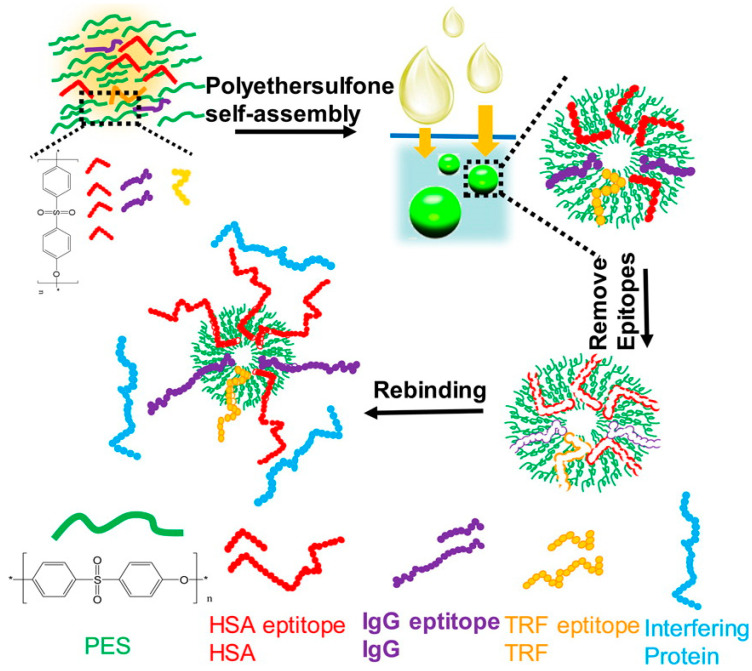
Fabrication of multiepitope-template imprinted particles via PES self-assembly [137].

In target substructure imprinting, epitope imprinting is the most widely used approach. Epitope imprinting has gained further development in recent years. Particularly, new approaches have been combined with epitope imprinting. Using bead-forming imprinting via mini-emulsion polymerization, L-lysine of immunoglobulin G was used as an epitope to fabricate imprinted beads to recognize immunoglobulin G [132]. To obtain higher imprinting efficiency and establish more important applications, a variety of epitope-imprinted polymers were synthesized extensively by surface imprinting [133,134,135,136,137]. Among these references, C-terminal nonapeptides of bovine serum album (BSA) [133,135] and N-terminal epitope nonapeptides of cytochrome C (Cyt C) [134,136] were used as templates to prepare MIPs specific to these target proteins. The above obtained MIPs were prepared by free radical polymerization or sol–gel reaction using different functional monomers. To develop a universal and straightforward strategy for epitope imprinting, an epitope from transferrin, the N-terminal nonapeptides sequence MRLAVGALL, was employed as the template to prepare MIPs on Fe_3_O_4_ nanoparticles (NPs) using a self-assembly method based on a polyethersulfone (PES) phase inversion [138]. The epitope-imprinted PES beads showed good selectivity, not only toward the epitope, but also toward target proteins. To achieve the simultaneous capture of various target proteins, Zhang and co-workers [137] further developed multiepitope-imprinted beads by phase inversion-based PES self-assembly. The N-terminal peptides of these three proteins, MKWVTFISL(N-terminal of HSA), ASTKGPSVF (N-terminal of IgG) and MRLAVGALL (N-terminal of TRF), were used as the epitope templates. The fabrication of multi-epitope-imprinted particles using PES self-assembly is demonstrated in Figure 12. PES and three epitope templates were dispersed in N, N-dimethylacetamide. The resulting polymer solution was added dropwise to distilled water at room temperature to fabricate the multiepitope-imprinted beads via phase inversion. After the epitope templates were dissociated, the imprinted particles obtained could capture the target proteins simultaneously with high selectivity, even in such a complex matrix as human plasma.

The epitope-imprinted NPs that were prepared exhibited specific recognition towards the target protein. In order to better control the capture and release processes, the researchers subsequently developed thermoresponsive epitope-oriented surface-imprinted NPs for specific capture and release of the target protein [139]. After the immobilization of a His-tag-anchored epitope of HSA, AASQAALGL-His-tag, the polymerization was conducted using N-isopropylacrylamide (NIPAAm) as the major monomer to form the thermosensitive imprinting layer. The resulting MIP showed high adsorption capacity and excellent selectivity toward the target protein compared to its non-imprinted counterpart. Moreover, the MIP demonstrated the capability to efficiently bind and release the desired protein from human plasma in a manner that is influenced by temperature. 

In the above works, metal ions did not participate in the rebinding with the targets. However, metal ions could be involved in the rebinding process. Zhang and co-workers [140] developed epitope-oriented surface-imprinted NPs using His-tag as a template for highly efficient purification of His-tagged proteins. The His-tag of His-tagged proteins was bound by the imprinted cavities, while other host proteins without a His-tag were excluded from the imprinted cavities. In this study, imidazole instead of EDTA was used to competitively elute the His-tag epitope, but not to release the immobilized metal ion (Ni^2+^). The participation of metal ions in imprinting cavities can greatly improve the affinity and selectivity of the epitope-oriented surface-imprinted NPs for His-tagged protein.

**Figure 13:**
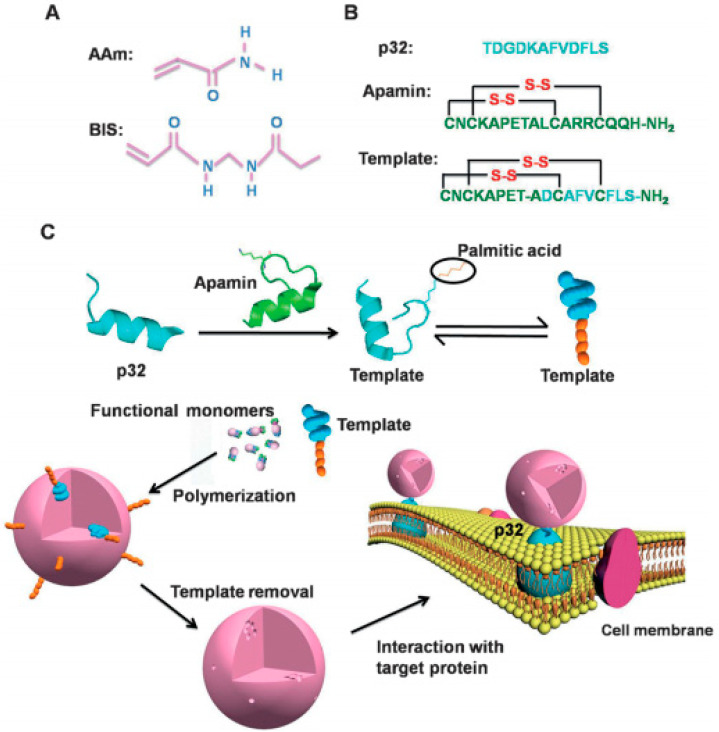
Molecularly imprinted polymeric nanoparticles designed for specifically recognizing a membrane protein [141]. (**A**): Monomer and cross-linker; (**B**): Template; (**C**): Surface molecular imprinting process.

The above mentioned epitope-imprinting methods mainly focused on the use of short linear peptides as epitope templates. In fact, most antibodies recognize a conformational epitope because of the epitope’s specific three-dimensional shape rather than its linear structure. In order to develop epitope-imprinted polymers with higher specificity for target proteins, Li and co-workers [141] used a disulfide-linked α-helix-containing peptide, apamin, to mimic the extracellular, structured N-terminal part of the protein p32 and then serve as an imprinting template for fabricating sub-40 nm-sized polymeric NPs using a combination of surface molecular imprinting and scaffold-based peptide design (Figure 13). The MIP nanoparticles were synthesized through inverse microemulsion polymerization, with hydrophilic AAm and MBAA serving as functional monomers due to the water solubility of the epitope template. The MIPs produced using the conformational epitope imprinting method offered precise targeting of membrane proteins, particularly those with conformational epitopes made up of noncontiguous sequences.

In addition to epitopes of proteins, some substructures or fragments of targets have been also employed as imprinting templates. In recent years, dihydrokainic acid, the peptide CEHRGWFNDC-K(N3), glycan, and sialic acid have been used in the synthesis of MIPs for the recognition of domoic acid [142], proteins [143], and cells [144]. The prepared MIPs exhibited satisfactory specificity for the targets and have found important applications. However, the binding affinity and binding selectivity of the MIPs obtained based on target substructure imprinting were relatively low.

### 3.4. Hierarchical Imprinting

**Figure 14:**
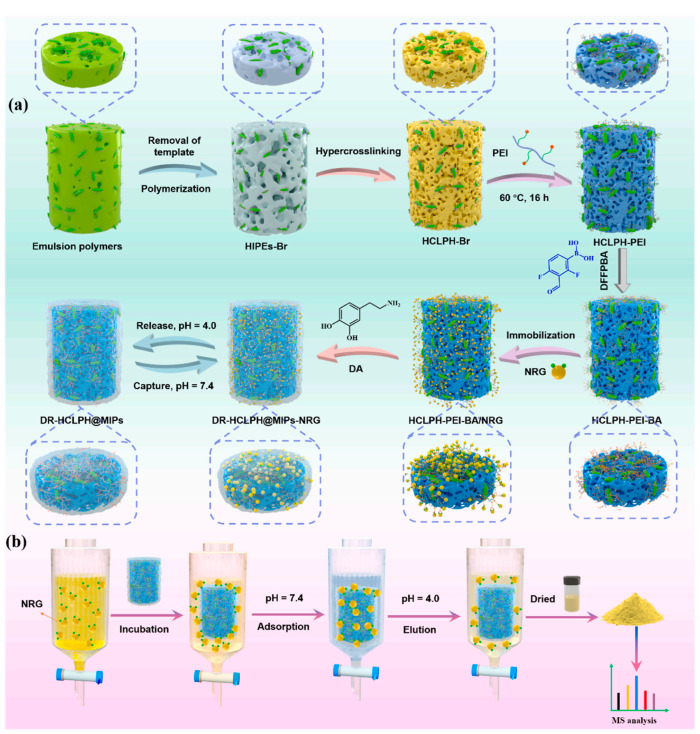
Schematic illustration of preparation process of DR-HCLPH@MIPs (**a**), and the selective separation process of DR-HCLPH@MIPs towards NRG (**b**) [145].

Hierarchical imprinting is a new surface imprinting technique. In general, a template is physically immobilized to a pore-generating agent such as a disposable porous solid. The polymerization of monomers in the pores of the pore-generating agent, followed by subsequent removal of the pore-generating agent and template, leads to the formation of a porous solid containing surface-confined template sites. As such, hierarchical imprinting can improve the site accessibility of the resultant MIPs and increase the efficiency of the imprinting process. Porous silica is the most commonly utilized mold for manipulating the particle size, shape, and porosity of the produced imprinted polymers. Recently, Liu and co-workers [145] developed double-imprinted anchor points in cellulose nanocrystals-based hierarchical porous polyHIPEs based on hyper-cross-linked PH (DR-HCLPH@MIPs) for flavonoid recognition and capture using the hierarchical imprinting technique. The principle of this approach is shown in Figure 14. The hierarchical porous HIPEs were stabilized, and then HCLPH-Br was obtained via HIPEs polymerization. Subsequently, the PEI was modified onto the surface of HCLPH-Br, and then the boronic acid monomer 2,4-difluoro-3-formylphenylboronic acid (DFFPBA) was modified onto the surface of HCLPH-PEI. After further pre-assembly of the boronic acid functional monomer DDFPBA with naringin (NRG), the dopamine monomer was introduced onto the HCLPH-PEI-BA/NRG to form DR-HCLPH@MIPs. This novel layer-by-layer assembly imprint method was adopted to construct DR-HCLPH@MIPs with several advantages, such as superb selectivity and rapid adsorption/elution rate toward template NRG molecules. Since then, hierarchical imprinting has been successfully applied in the preparation of other protein imprinted materials [146,147,148]. These hierarchically imprinted polymer beads have been shown to recognize proteins with excellent selectivity, and have high binding capacities and fast mass transfer capacity. In addition, the epitope imprinting strategy has been combined with the hierarchical imprinting approach to prepare MIPs for the separation of proteins and peptides. Dong and colleagues developed hierarchically imprinted polymers for the specific separation of lysine acetylated peptides. KacA, a dipeptide containing acetylated lysine and alanine residues, was used as the template and immobilized onto a sacrificial silica support. The hierarchically imprinted polymers showed selectivity for Kac-peptides with different molecular sizes, indicating the involvement of epitope binding. The obtained MIPs also exhibited good extraction selectivity towards lysine acetylated peptides in the histone digest and whole cell lysates.

**Figure 15:**
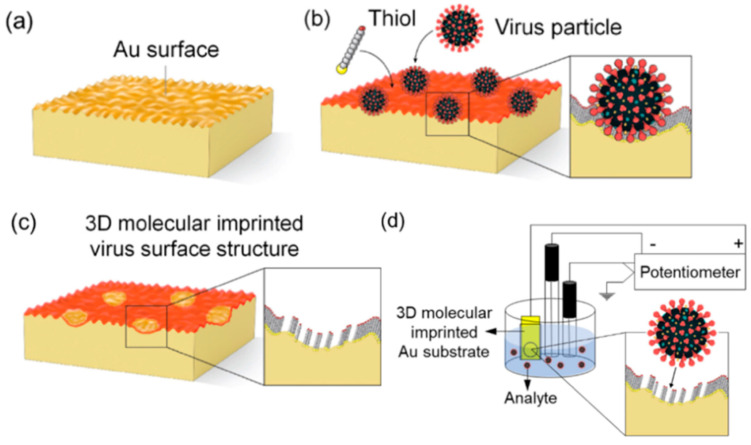
Schematic illustration of the 3D molecular imprinting process: (**a**) Au-coated surface showing the underlying roughness of the unpolished Si wafer and (**b**) imprinting process where thiols and template molecules (target analytes; virus particles in this case) are adsorbed onto the Au surface. The thiols are permanently bound into a SAM that crystallizes around the template molecules. (**c**) Template molecules, which are weakly adsorbed on the Au surface, are removed by washing in a 3 M NaCl solution, leaving behind imprinted areas in the SAM with an inverse replica of the surface molecular structure of the template molecules. (**d**) Potentiometric analyte detection: when the analytes are re-adsorbed into the imprinted SAM layer, it creates a change in OCP [149].

In recent years, molecular imprinting in monolayers has grown rapidly. In addition to being successful using common template molecules, the imprinting of whole virions and isolated proteins using this method has also been reported [149]. As depicted in Figure 15, OH-terminated thiols are adsorbed onto a Au-coated Si chip together with template molecules virions. The template molecules, being larger than thiols, adsorb first onto the Au surface and fit conformally onto the surface structure. At the same time, the thiols adsorb around the template molecules, reacting with the Au surface via Au–sulfur bonds, and crystallize into a 2 nm thick self-assembled monolayer (SAM). After removing the template molecules from the surface with a 3 M NaCl solution, an imprinted thiol layer is formed, which precisely mirrors the molecular structure of the original template on the surface. The imprinted Au chip then serves as an electrode of potentiometric analyte detection. In addition, Wattanakit and co-workers [150] developed chiral-imprinted mesoporous platinum electrodes for highly enantioselective synthesis based on controlled formation of a self-assembled monolayer.

### 3.5. Computer-Aided Imprinting

**Figure 16:**
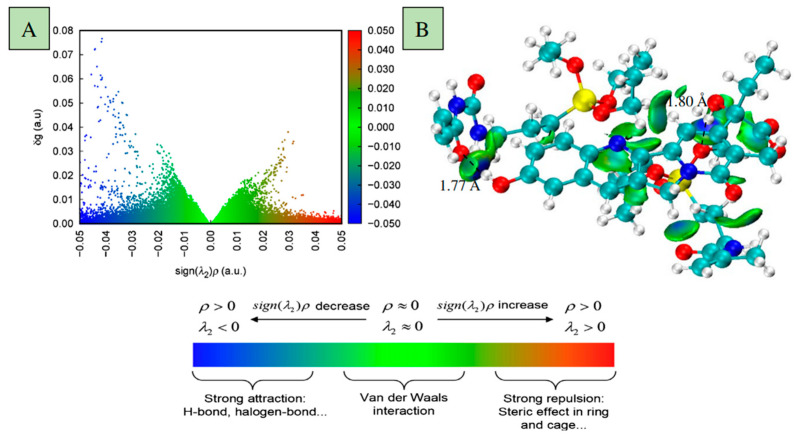
Scatter diagram of the δg vs. sign(λ_2_)ρ (**A**) and the gradient isosurfaces (**B**, s = 0.5 au) for the optimal complex [151].

It is worthwhile to mention that in the preparation of MIPs, the selection of a suitable functional monomer is a crucial step. However, in the traditional fabrication of MIPs, the selection of a functional monomer is based on experimental or empirical methods, which are inefficient and time-consuming. In addition, these methods tend to consume significant quantities of solvents, reagents, and labor. Computer simulations can be utilized for in-depth exploration of molecular structures and behaviors to predict the physical and chemical properties of molecularly imprinted polymers (MIPs). As early as 2015, Liu and colleagues used computer simulations to investigate MIP systems using enrofloxacin (ENRO) as the template molecule and various functional monomers, such as 2-vinyl-4,6-diamino-1,3,5-triazine(VDAT), 4-vinylpyridine(4-Vpy), acrylamide(AM), or trifluoromethacrylic acid(TFMAA). The density functional theory (DFT) was used to identify the optimized geometries of these systems, the optimal molar ratios of templates to functional monomers, and the active sites in the systems. Recently, by means of computer-aided technology, hydroxycamptothecin (HCPT) hybrid MIPs (AT/MA-HMIPs) with high selectivity and a hard silicon skeleton were successfully prepared based on double hybrid monomers [151]. The maximum adsorption capacity was 18.79 mg/g, and the binding equilibrium was within 20 min. These MIPs also showed excellent selectivity, with an imprinting factor of 10.73, and good recoverability for HCPT: after 10 adsorption-desorption cycles, the adsorption capacity had only decreased by 7.75%. As depicted in Figure 16A, it can be seen that there are dense gradient peaks at the negative position with a high g value, indicating that the hydrogen bond interaction between HCPT and AMTEOSPI, MAA-APTES was stronger, and that the binding system comprising the template molecules and functional monomers was stable. Figure 16B exhibits the gradient isosurface (s = 0.5 au), which displays the location where the interaction occurred.

## 4. Applications of Non-Covalently Imprinted Polymers

Due to the invention of the new imprinting approaches, many molecularly imprinted materials with desirable properties and in favorable material forms have been prepared within the past five years. These desired properties and material forms have enabled a variety of important applications, including affinity separation, chemical sensing, disease diagnostics, proteomics, bioimaging, controlled drug release, and catalysis. Particularly, MIPs have already exhibited great potential as important substitutes for antibodies for applications in disease diagnostics, proteomics, and bioimaging.

### 4.1. Affinity Separation

Due to the high selectivity of MIPs for target molecules, MIPs have been widely used as selective sorbents in the affinity separation of various compounds in real complex samples, such as environmental water [152,153,154,155,156], food [157,158,159], plants [160,161], egg white samples [162,163,164,165], and urine [166,167].

Recently, MIPs have been successfully applied in the selective separation and purification of harmful substances from food. Hu and co-workers [157] employed a cyromazine-imprinted polymer coated stir bar for the efficient extraction of melamine in milk. Due to the uniform and porous surface of the MIP-coated stir bar, along with its excellent chemical stability and selectivity for melamine, efficient extraction of melamine from milk samples was achieved. The recovery rate of melamine in the spiked sample was 65.0–111%. Additionally, MIP-based magnetic separation has been successfully used to specifically extract 17b-estradiol from milk, with a recovery rate of 88.9–92.1% [158]. In addition to harmful substances, more recently, MIPs have been successfully applied to the selective separation of drugs from food. Liu and co-workers [159] have provided a theoretical foundation for the application of aerogel-based molecularly imprinted polymers in oleanolic acid-targeted separation in the fields of medicine or healthy food products.

MIPs have also been successfully applied in the isolation of chicoric acid from *Chicorium intybus* L., a medicinal plant [159]. Recently, Zhang and co-workers [161] developed the molecularly imprinted membrane electrospray ionization (MIM-ESI) technique for rapidly detecting four classes of pesticide residues in Chinese herbal medicines, including organophosphorus, carbamates, pyrethroids, and neonicotinoids. The optimal experimental conditions were determined, and the method was further validated for high sensitivity and specificity. The data showed that MIM-ESI MS is applicable for the direct quantitation of pesticide residues in Chinese herbal medicines.

Due to excellent specificity and imprinting efficiency, imprinted magnetic carbon nanotubes [162], polydopamine-based NIR-light responsive imprinted nanofibrous membranes [163], UiO 66-based molecularly imprinted polymers [164], and surface-imprinted core-shell nanorods [165] have been successfully used to separate non-glycoproteins and glycoproteins from fresh egg white samples, respectively.

Fluoroquinolones (FQs) belong to a group of synthetic broad-spectrum antibiotic medications. By inhibiting mRNA transcription and DNA replication, FQs are effective against both Gram-positive and Gram-negative bacteria. In order to assess FQs in human urine, Fe_3_O_4_@MIPs NPs were applied in the highly selective magnetic separation of fluoroquinolones in human urine [166]. For FQ-spiked human urine samples, satisfactory extraction was achieved; the recovery rates were 83.1–103.1% with an RSD of 0.8–8.2% (n = 3). Furthermore, MIPs could be used for the selective extraction of cotinine in human urine [167].

### 4.2. Chemical Sensing

In the past decade, there has been a growing utilization of MIPs in the advancement of chemo/biosensors owing to their favorable attributes, like high specificity, stability, affordability, and ease of preparation. The key aspect of biomimetic sensors lies in establishing a dependable connection between the target binding event and the transducer. Consequently, a significant challenge in developing MIP-based sensors is determining how to quantify analyte binding on MIP materials. Typically, MIP-based sensors are created by attaching MIP material onto a transducer surface, which converts analyte binding into a detectable signal. Ultimately, the effectiveness of sensors hinges not only on the selectivity and sensitivity of MIPs to the target species, but also on the methodologies of signal output. A highly effective transduction strategy for producing readable signal outputs should aim to enhance both the selectivity and sensitivity of sensors.

#### 4.2.1. Electrochemical Sensor

Electrochemical biosensors combine the sensitivity of electroanalytical methods with the inherent selectivity of the biological component [168]. MIP-based electrochemical sensors were first reported in the early 1990s by Mosbach‘s group [169]. Obtaining satisfactory quantitative analysis results is dependent on various factors that affect the performance of electrochemical MIP biometric sensors, such as nature of analytes, the electrochemical probes used, and the specificity and affinity of the MIPs. In terms of signal response, electrochemical sensors can be classified into the following three major types: (1) electric current (amperometry and voltammetry), (2) potentiometry (ion-selective electrodes (ISEs) and field-effect transistors (FETs)), and (3) capacitance/impedance.

**Figure 17:**
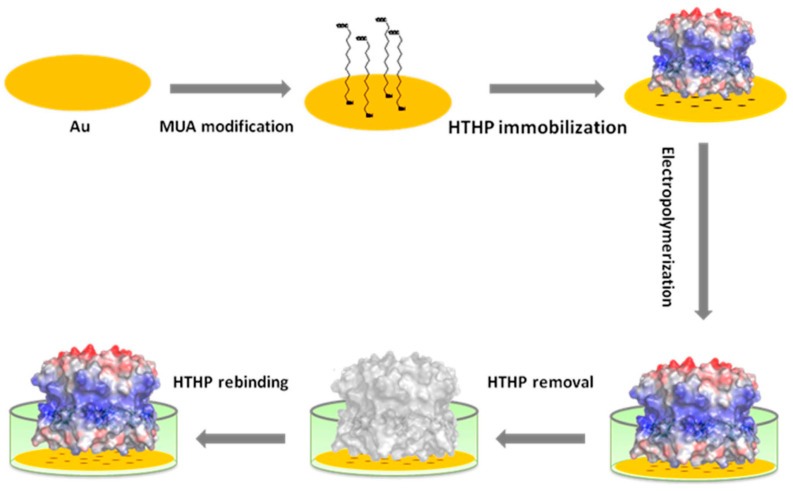
Schematic representation of the MIP preparation on a negatively charged thiol terminated SAM. Red: negatively charged region of HTHP, blue: positive region [171].

Amperometry sensors can detect electroactive species directly by establishing a linear relationship between the concentration of electroactive species and the current measured at constant potential [170]. Hybrids composed of a biocatalyst and a MIP may combine the advantages of both components. This combination can improve selectivity in sensors that operate on the basis of either a “group-specific” enzyme or a binding MIP. Recently, Scheller and co-workers [171] developed a molecularly imprinted electropolymer for a hexameric heme protein with direct electron transfer (DET) and the bioelectrocatalytic activity of the target protein. As depicted in Figure 17, the electrochemical MIP preparation involved its insertion into 0.5 mM scopoletin solution containing 5 mM NaCl. The low ionic strength was used in order to prevent the dissociation of hexameric tyrosine-coordinated heme protein (HTHP) from the SAM. In this process, scopoletin was polymerized and formed a network around the template HTHP. Scopoletin was chosen as the monomer because in previous work we succeeded in the preparation of MIPs for Cyt C and the lectin concanavalin A (ConA) by electropolymerizing it on top of a SAM. The catalytic current showed a linear increase as the concentration of H_2_O_2_ increased from 10 to 100 µM, reaching saturation at 150 µM. To electrochemically detect a non-electroactive target using a MIP-based amperometry sensor, a new electrochemical sensing receptor based on the introduction of a redox tracer inside the binding cavities of cross-linked MIPs was developed [172]. Such an electrochemical MIP (e-MIP) can serve both as the recognition and the measuring element for the electrochemical determination of a non-electroactive target. The e-MIP was prepared in a simple and conventional way by copolymerization of a functional monomer presenting electroactive properties with a cross-linker. The analyte (benzo[a]pyrene) was successfully determined by simply measuring the redox tracer signal. Amperometric sensors have been considered to be the most convenient electrochemical sensing platform due to their low cost and easily adaptable instrumental setup.

Voltammetry is the most frequently used approach in MIP surface characterization due to its intrinsic redox potential properties. Various types of voltametric techniques have been applied to characterize MIP surfaces for selective detection of analytes. Commonly used voltametric techniques include linear sweep voltammetry (LSV) [173], cyclic voltammetry (CV) [174], differential pulse voltammetry (DPV) [175], square wave voltammetry (SWV) [176], and anodic stripping voltammetry (ASV). For LSV and CV, potentials linearly change, while DPV and SWV are characterized by a continuous increase in either rectangular pulse or square oscillations, which can offer better sensitivity and signal-to-noise ratios than ASV and CV [170]. Due to their high specific surface area, nanostructural materials used in electrochemical sensors can significantly increase the selective surface area and lead to larger numbers of imprinted sites for improved sensitivity by enhanced electron transfer. Thus, the use of core-shell molecularly-imprinted NPs in electrochemical sensors is considered highly feasible. For example, Tang and co-workers [177] introduced an electrochemical sensor based on the in situ electropolymerization of a pyrrole-modified glassy carbon electrode. This electropolymerized polypyrrole MIP-modified glassy carbon electrode was used in the determination of butachlor by DPV. Under optimized conditions, the developed sensor provided a rapid and stable linear response to butachlor between 0.107 and 500 ng L^−1^. A satisfactory limit of detection of butachlor was 0.0491 ng L^-1^. The sensor was effectively utilized in water samples, achieving a satisfactory recovery rate ranging from 98.4% to 102%.

MIPs have been used to develop selective or specific potentiometry sensors. In MIP-based potentiometry, a flow stream potential across the column electrode with varying concentrations of target analyte was continuously recorded. Two basic types of potentiometric sensors, including ion-selective electrodes (ISEs) and field-effect transistors (FETs), have been adopted as transducers for measuring the recognition features of MIPs. The obtained MIP-based potentiometry sensors have been used for the recognition of electroactive species, such as ions and biomolecules. Recently, ion-selective electrodes (ISEs) coated with molecularly imprinted NPs synthesized via solid-phase imprinting were applied in the potentiometric detection of histamine, which gave a limit of detection (LOD) of 1.12 μmol/L and a linear range between 10^−6^ and 10^−2^ molL^−1^ [178]. The sensor was also successfully used to quantify histamine in wine samples. The LOD of histamine in wine samples was calculated at 8.05 × 10^−6^ mol/L and 1.69 × 10^−4^ in 1:5 and 1:10 dilutions, respectively. Direct measurements of non-diluted wine led to a higher LOD (2.57 × 10^−4^ mol/L), unsuitable for histamine analysis in wine. Consequently, higher wine dilutions allowed for better histamine sensing due to reduced interferences, but the dilution itself hindered proper detection. In this regard, a 1:5 wine dilution was selected, since it was found to be able to provide the best compromise between detection of histamine and reduction of matrix effect. Recently, Gyurcsányi and co-workers [179] developed 2,2,6,6-tetramethylpiperidin-1-yl)oxyl (EMPO)-functionalized carbon nanotubes for solid-contact ion-selective electrodes (SCISEs) with largely improved potential reproducibility and stability. SCISEs without prior conditioning could be applied for accurate K + measurements in undiluted blood serum. However, detection of uncharged non-electroactive/neutral molecular species by MIP-based potentiometric sensors still remains an open challenge.

Recently, the use of FET was reported in the selective determination of inosine by an inosine-templated MIPs film deposited on an extended gate field-effect transistor (EG-FET) signal transducing unit. This served as a recognition and signal transduction unit, providing high sensitivity to the integrated chemosensor device. The linear dynamic concentration range was 0.5–50 μM, with a high detectability of 0.62 μM. The obtained detectability compares well to the levels of the inosine in body fluids, which are in the range 0–2.9 μM for patients with diagnosed diabetic nephropathy, gout, or hyperuricemia, and can reach 25 μM in certain cases. Moreover, the MIP film-coated transistor based chemosensor showed advantageous flexibility during measurements through gate voltage adjustments, granting it potential as a promising tool for devising and developing MIP-based FETs. In addition, extended-gate field-effect transistor (EG-FET)-based chemosensors with molecularly imprinted polymers (MIPs) have also been employed in the selective determination of tumor necrosis factor-α (TNF-α) in serum [180]. MIP-based EG-FET sensors applied in this context exhibited high sensitivity, with a LOD of 0.55 pg/mL and excellent selectivity (coefficient(α) > 3). Based on this excellent sensing performance, comprising high sensitivity and selectivity as well as excellent reproducibility and reusability, this (EG-FET)-based chemosensor with TNF-α-recognizing MIP film can be used for the early diagnosis and point-of-care of immune-related diseases.

MIPs have been used to develop selective or specific impedance or capacitance sensors. Impedance/capacitance sensors are another type of electrochemical sensor that involve the insulation of MIP-based film to provide recognition. Due to the electrical double-layer structure formation, such sensors have several merits, such as high sensitivity, being label-free, and having the capability for real-time monitoring of target analytes [181]. Roy and co-workers [182] prepared a molecular-imprinted bipolymer to construct a new capacitive sensor for the direct detection of inositol in fruits. The linearity range of the sensor was from 0.125 to 1 ppm, and the LOD was estimated to be as low as 1.8 ppb. Linear regression models, including the principal component regression (PCR) method and partial least square regression (PLSR), were used for the prediction of IS in the fruits. The prediction accuracies were found to be as high as 99.91% and 99.90% for the two methods, respectively. The root mean square error of calibration (RMSEC) was estimated to be 0.0008 for both PLSR and PCR using three latent variables.

In conclusion, electrochemical sensors have been successfully functionalized with molecularly imprinted NPs or films, which allow for online monitoring of drugs and contaminants in pharmaceutical and medical samples, as well as in food matrices. MIP-enhanced electrochemical sensors have the benefits of cost-effectiveness, simple preparation, robustness, miniaturization possibility, and fast response. However, there are many considerations that need to be made in the design and manufacture of these sensors, including materials, costs, and manufacturing methods that are compatible with mass fabrication and real-sample application. Furthermore, these sensors need to be useful in a complex environment and in real-world sample matrices; otherwise, they will fail in commercial application.

#### 4.2.2. Optical Sensors

Optical sensors combine the sensitivity of optical methods with the inherent selectivity of MIPs. MIP-based optical sensors can be mainly classified into the following two types, based on their signal response: fluorescence sensor and surface-enhanced Raman scattering.

Direct fluorescence sensors detect analytes with intrinsic fluorescence. They require the analyte to have at least one fluorophore that can provide transduction signals for qualitative and quantitative analysis. Moreno-Bondi et al. [183] developed nano-patterned rhodamine 123 (R123)-MIP film arrays by electron beam lithography (EBL). The MIP film arrays showed strong selectivity towards R123 and demonstrated positive-tone behavior within the dose range of 0.1–8 mC cm^−2^. This study produced new opportunities in the implementation of nanostructured MIP film-based arrays for direct target detection. However, only a limited number of compounds emit intrinsic fluorescence, and thus a considerable demand for fluorescent dyes has been triggered in order to facilitate the preparation of MIPs with fluorescence. To this end, various strategies have been developed to incorporate a suitable fluorescent dye into the MIP network to monitor non-fluorescent analytes.

Typically, a fluorescent molecularly imprinted polymer (MIP) is necessary for indirectly detecting a non-fluorescent analyte. Rurack and co-workers [184] reported a “light-up” type of fluorescent sensor consisting of a silica core coated with a MIP shell. The MIP layer contains a fluorescent probe cross-linker which binds selectively to phosphorylated tyrosine moieties with a significant imprinting factor (IF) and responds with a “light-up” fluorescence signal. The sensor exhibited the detection of peptides with N-terminating phosphorylated tyrosine.

In addition, noble metal NPs (such as AgNPs and AuNPs) could enable dramatic changes in fluorescence signals, potentially driving the detection limit to below the nM level. Therefore, the integration of NPs and MIPs has been utilized in targeted sensing applications. A magnetite core-shell material with gold nanoclusters prepared using molecular imprinting techniques has been successfully synthesized [185]. Gold nanoclusters (AuNCs) and MIPs were used to prompt changes in the fluorescence intensity when bisphenol A (BPA) was bound to the prepared core-shell material. The sensor was used for fluorescence detection of bisphenol A in aqueous solution.

Another class of fluorescent nanomaterials includes quantum dots (QDs), which have size-dependent emission wavelengths, high luminescence efficiency, and good photostability. The high selectivity of MIPs combined with the high sensitivity of QDs has been utilized to reduce the LOD and analyze trace substances in samples. CdSe/ZnS QDs were coated with a MIP shell to selectively detect aminoimidazole-azaarenes (AIAs) [186] through fluorescence quenching with increasing analyte concentration. The LOD was 0.25 mu g L^−1^ (3 sigma/s). Moreover, fluorescent materials have been further applied to determine AIAs in beef floss and grilled fish fillet with satisfactory recovery rates (82.8–110.8%) and a relative standard deviation (RSD) of <5.5%. Silica NPs can act as a support material due to their high chemical and mechanical stability. One example used silica NPs as a support to immobilize CdTe QDs coated with a thin MIP layer to detect bovine serum albumin in bovine calf serum samples [187]. The MIP-coated QDs exhibited distinctly linear fluorescent quenching toward BSA in the concentration range of 5.0 × 10^−7^ to 1.0 × 10^−5^ M with a correlation coefficient of 0.9957 and a LOD of 1.1 × 10^−7^ M. Optical sensors based on QDs have gained interest recently due to their intrinsic optical properties, such as wide absorption spectra and narrow emission, which make them promising candidates for optical detection of various analytes. When comparing a QD-based immunosensor with a QD-MIP-based sensor, the latter offers advantages such as enhanced chemical stability, cost-effectiveness, and the capability to detect small molecules.

Furthermore, sensor arrays using synthetic receptors in molecularly imprinted polymers have achieved increased accuracy and the added benefit of customizable specificity through the molecular imprinting process. Niu and co-workers [188] constructed biomass carbon dots–MIP fluorescent sensor arrays for the accurate identification of 5-nitroimidazole antibiotics. This fluorescence sensor array can sensitively identify these 5-NDZs in a wide range (20–5000 nM). In order to develop a novel imprinted photoelectrochemical sensor, difunctional MIPs and heterostructured CdS nanoparticle-sensitized ZnO nanorod sensor arrays were developed for alpha-fetoprotein (AFP) detection [189]. The prepared sensor had a wide linearity in the range from 1 pgmL^−1^ to 1000 ng mL^−1^, with a low LOD of 0.38 pg mL^−1^. Additionally, a set of four-channel sensor arrays was constructed using carbon dots (CDs) embedded in photonic crystal molecularly imprinted (PCMIP@CDs) film [190]. The sensor array achieved 100% accuracy in classifying five sulfonamides (SAs) in water and fish samples.

Surface-enhanced Raman scattering (SERS) has garnered considerable interest due to its numerous advantages, including its ultra-high sensitivity, resilience to sample environment variations, and potential for on-site or field detection when compared to fluorescence detection. The combination of a MIP with SERS is very attractive because a MIP can provide high specificity, while SERS can provide high sensitivity. This combination’s application has been limited due to issues like the high reactivity and instability of SERS-active surfaces under ambient conditions, though MIPs have been created to monitor the release and uptake of N-benzyloxycarbonyl-(L)-aspartic acid in aqueous solution. Lu and colleagues worked on the development of a MIP-based SERS sensor for detecting melamine in whole milk, utilizing silver dendrite nanostructures as the SERS-active substrate to generate signals. Prior to the detection of melamine in raw milk samples, effective clean-up of whole milk samples was achieved using MIPs as extraction sorbents. SERS permitted rapid and sensitive detection of melamine, providing a LOD of 12 μM and a limit of quantification (LOQ) of 39 μM. Although these MIP-based SERS sensing methods provided good detection platforms for real sample applications to some extent, the sensitivity and stability of the MIP-based SERS substrates still requires further improvement. Indeed, recent progress has been made in rendering MIP-based SERS sensors more applicable for food quality testing. Khatoon and her team developed a molecularly imprinted polymer sensor for highly sensitive norfloxacin detection using SERS technology. As a result, a LOD of 2.5 × 10^−11^ M for the target molecule norfloxacin was achieved, which is 2–3 orders of magnitude more sensitive than that stipulated by European Union regulation (8.0 × 10^−9^–4.0 × 10^−8^ M). Such SERS-based MIPs show great promise in the separation and detection of trace amounts of antibiotics and their residues in environmental samples like tap water and milk. Liao and co-workers [191] proposed a simple synthesis route for molecularly imprinted 3D SERS substrate with inorganic frameworks for the selective detection of rhodamine 6G (R6G). The prepared SERS sensor enabled sensitive and selective detection of R6G in food samples with a LOD of 0.27 nM. SERS is regarded as a promising candidate for the quantification of target molecules in foodstuffs. However, prior to developing a detection assay, the key parameters which are essential for the establishment of the method need to be investigated, such as the accuracy and precision of the method, its selectivity among similarly structured molecules, and its cross-reactivity during the fabrication of the biosensor.

**Figure 18:**
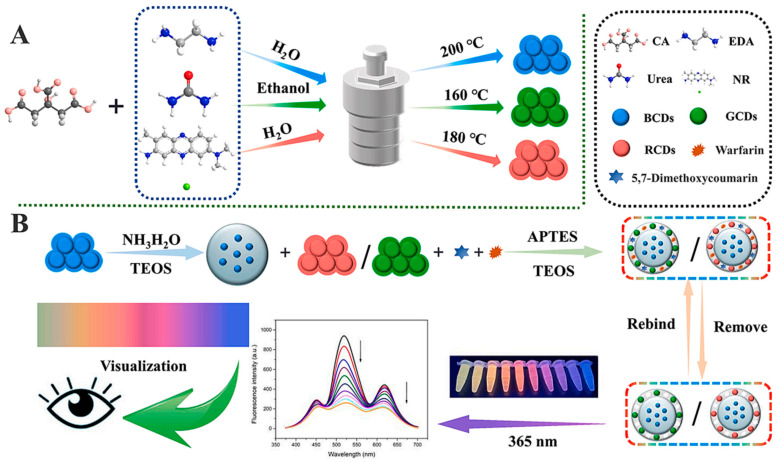
Schematic illustration of (**A**) Synthesis of three-emission carbon quantum dots; (**B**) the preparation and detection process of the proposed sensor [192].

It is becoming more important that new MIP-based sensors should be easy to use, inexpensive, portable, and if possible, allow detection by the naked eye. By and large, to achieve naked-eye detection, unique signal amplification and transduction strategies are required. Thanks to the rapid development in the field of macromolecules, conjugated hydrogels have become an ideal substrate for the above purpose. As an illustration, Spivak and colleagues in 2006 created a dual-imprinted diffraction-grating sensor for detecting viruses. By employing a simple and automatic laser transmission apparatus, the MIPs were incorporated into a multi-array substrate for fast and large-scale measurements. This system can be read by the naked eye to detect the apple stem pitting virus (ASPV) at concentrations as low as 10 ng/mL. An additional novel aspect of this bioimprinting study is that it is the first example of the use of an impure virus extract as the source of the template, an approach that was facilitated by the use of virus-specific aptamers that excluded interference from the other extract components in the pre-polymerization complex. Based on this novel technique, Liu and co-workers [192] presented a novel type of dual-template molecularly imprinted polymer ratiometric fluorescence sensor based on three-emission carbon quantum dots (CDs) for accurate naked-eye detection of aflatoxin B1 and zearalenone in vegetable oil (Figure 18). In this process, the three-emission CDs in Figure 18A were first prepared by the hydrothermal reaction in different solvents and temperature, indicated by for arrow and different color lines. The blue CDs (BCDs)-embedded silica nanoparticles (BCDs@SiO_2_) were then obtained by the Stober method. Under optimized experimental conditions, aflatoxin B1 and zearalenone exhibited satisfactory linearity at concentration ranges of 0.01–100 ng/mL and 0.03–100 ng/mL, with LOD values of 3.2 pg/mL and 18 pg/mL, respectively.

#### 4.2.3. Mass Sensitive Sensors

Mass-sensitive sensors consist of weight signal units such as piezoelectric devices and quartz crystal microbalances (QCMs). QCMs have been widely used as transducers to develop MIP-based biosensors, not only for single-analyte detection, but also for multi-channel and multi-analyte detection. This label-free technique is particularly well suited to macromolecular analytes, such as proteins, and biological particles, such as viruses and cells, due to their large mass. In MIP-based QCM sensors, quartz crystal resonators are usually integrated with MIPs to measure either mass changes or increased damping when analyte molecules are adsorbed onto the surfaces of the sensors. Denizli and colleagues introduced a label-free, rapid, and selective detection technique by utilizing micro contact imprinting of whole *Escherichia coli* (*E. coli*) cells on both optical (SPR) and mass sensitive (QCM) devices. Detection of *E. coli* in apple juice by this label-free approach was demonstrated. Recently, several new MIP-based QCM sensors have been reported. A novel molecularly imprinted QCM sensor based on a molybdenum disulfide nanoparticle (MoS2NPs)-multiwalled carbon nanotube (MWCNT) nanocomposite was prepared for the selective determination of zearalenone in rice samples [193]. A linear relationship was observed between the frequency response of the sensor and the concentration of injected analyte over the range 1.0–10.0 ng L^−1^. The LOD was evaluated to be 0.30 ng L^−1^.The developed sensor’s high repeatability, reusability, selectivity, and stability allowed for the dependable detection of zearalenone in rice samples.

### 4.3. Disease Diagnostics

With the rapid development of proteomics and metabolomics, potential disease biomarkers can be discovered from human tissues and organs. Biomarkers play a key role in disease diagnosis and prognosis [194]. Thus, a reliable and sensitive detection approach for these biomarkers is of great importance in practical applications. Immunoassays, which rely on the use of antibodies, are a general method used to diagnose biomarkers. However, antibodies are hard to prepare, expensive, and unstable during storage. Therefore, inexpensive and stable alternatives to antibodies have not only scientific significance, but also considerable prospects in practical application. Recently, MIPs have been used as efficient binding tools for the quantitative detection of disease biomarkers in disease diagnostics. Enzyme-linked immunosorbent assays (ELISA) have long been an important approach for disease diagnostics [194]. However, MIPs, as a substitute for antigens and antibodies, have also been used as recognition units, due to their low cost and high chemical stability. MIPs can be directly polymerized at the bottom of a microplate or grafted on the base of a well after external polymerization [195]. Different synthesis strategies and target biomarkers can be used, depending on the target analysis [195,196]. On the other hand, the lack of reproducibility and the difficulty involved in coating microplate wells makes their use unfeasible in quantitative analyses.

### 4.4. Proteomics

A proteome is the entire set of proteins expressed by the genome in an organism or a biological system. Reversible protein phosphorylation and glycosylation are two key events in numerous biological processes, and have been recognized as two of the most popular post-translational modifications. Phosphoproteomics and glycoproteomics have become hot research topics because they are vital for unraveling biological processes and finding new strategies for the diagnosis and treatment of diseases [197]. The study of phosphoproteomics mainly focuses on the identification of phosphorylation sites, site occupancy, and quantitative analysis of phosphoproteomics, while the study of glycoproteomics mainly focuses on the identification of glycosylation sites, glycan structure analysis, site occupancy, glycan isoform distribution, and quantitative analysis of the glycoproteome. MS is the most powerful and essential tool in proteomic research studies, since MS can provide both structural and quantitative information. However, direct MS-based phosphoproteomics and glycoproteomics analysis still faces great challenges. This is mainly due to the fact that glycopeptides or phosphopeptides have relatively low abundance, and their ionization efficiency is much lower compared with non-glycopeptides or non-phosphopeptides; thus, their signal can be significantly suppressed by the presence of other, highly abundant components. Therefore, highly efficient enrichment strategies are required to isolate and enrich phosphopeptides and glycopeptides prior to MS analysis.

**Figure 19:**
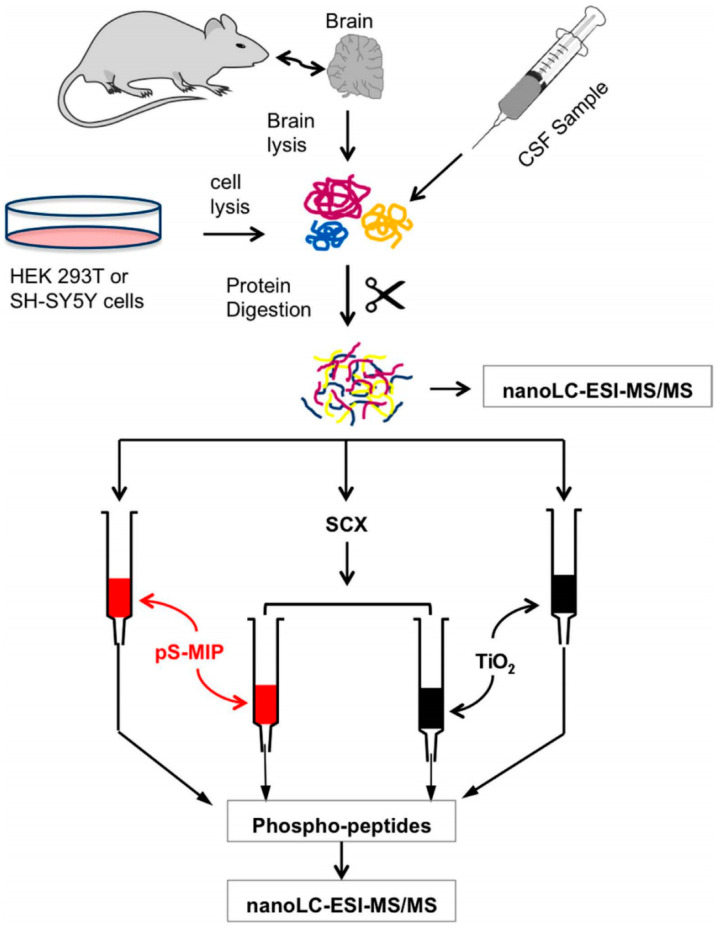
Workflow for phosphoproteomic analysis of harvested HEK 293T cells, mouse brain, or CSF using SCX fractionation followed by pS-MIP or TiO_2_ enrichment. Samples (10 μg) of cell lysate or CSF tryptic digests were loaded before or after pre-fractionation with SCX onto pS-MIPs or TiO_2_ columns for phosphospecific enrichment [199].

For phosphoproteomics, immunoaffinity and chemical modifications have been the most commonly used strategies for the selective enrichment of phosphopeptides and phosphoproteins from complex samples [198]. However, these strategies suffer from unavoidable problems such as high-cost antibodies, limited general applicability, and the degradation of biosamples. These limitations have been overcome by MIP-based affinity materials in recent years. There are four different types of phosphoamino acid residues: O-phosphates (O-phosphomonoesters), N-phosphates (phosphoamidates), acylphosphates (phosphate anhydrides) and S-phosphates (S-phosphothioesters). Among them, O-phosphates, which are mostly attached to serine, threonine, and tyrosine residues, are by far the most abundant. Phosphorylation on Ser and Thr residues occurs more frequently than on Tyr residues [199]. One study used pSer-imprinted polymers to enrich endogenous phosphopeptides from HEK 293T or SH-SY5Y cells. Cultured HEK 293T cells were harvested, lysed, and the proteins were trypsinized into peptides. These peptides were then fractionated using four different phospho-selective solid-phase extraction (SPE) protocols involving pSer-MIP, SCX, and/or TiO2 phases, as illustrated in Figure 19. After 10 μg of digested HEK 293T cell lysate was enriched by pSer-MIP, the phosphopeptide number increased to 237. However, following pre-fractionation of trypsinized proteins by strong cation exchange (SCX) chromatograph, pSer-MIP enrichment led to the identification of 924 phosphopeptides in the HEK 293T cell lysate, exceeding the number identified by TiO_2_-based enrichment (230). To assess the effectiveness of the pSer-MIP approach in different cell types, a tryptic digest of the human neuroblastoma cell line SH-SY5Y was analyzed. In this scenario, 648 phosphopeptides were identified solely through the use of pS-MIPs. By adding SCX pre-fractionation, 1271 phosphopeptides were identified, a number greater than that obtained using HEK 293T cells. In addition, pSer-MIP can also be applied to scarce neurological research samples. Applying the method to human CSF led to the discovery of 47 phosphopeptides from 24 phosphoproteins and revealed three previously unknown phosphorylation sites, among which 8 phosphoproteins with a total of 19 phosphopeptides in human CSF were reported for the first time. Very recently, Huynh and co-workers [200] described the manufacture of monolithic MIPs prepared by a step-growth polymerization process. The MIP capillary column was able to separate monophosphorylated peptides from a tryptic digest of bovine serum albumin.

### 4.5. Bioimaging

Selective cell recognition and capture has recently attracted significant interest due to its potential importance in clinical, diagnostic, environmental, and security applications. Cancer, one of the deadliest illnesses throughout the world, is diagnosed in millions of people annually, according to different cancer controlling government agencies. Whilst significant progress in cancer treatment has been made in recent years, oncologists suggest that early detection and disease progression monitoring would save many lives. Cancer cells physically and chemically differ from normal cells and thus are easy to differentiate. However, their low abundance and heterogeneity (variations in cell shape, size, and surface), depending on the origin, location, and stage of the illness, can make early detection difficult. To meet the pressing need for early detection and monitoring of disease progression, the design of materials and surfaces capable of the selective recognition and capture of living cells has attracted great research interest from the scientific community in the past few years. Most reported strategies for cellular imaging rely on specific antibodies. Unfortunately, antibodies suffer from disadvantages such as poor availability and stability, as mentioned above. MIP NPs can be used as a potential alternative to antibodies for bioimaging because they can provide antibody-like binding properties. MIPs are potentially superior receptor materials for cell imaging applications for a number of reasons: they are physically and chemically very stable and are not degraded by proteases or denatured by solvents, and they can be easily functionalized with fluorescence dyes, quantum dots, and even SERS report molecules.

In 2015, Haupt and colleagues successfully synthesized fluorescently labeled glucuronic acid-imprinted nanoparticles and utilized them for the first time in cellular and tissue molecular imaging to pinpoint and measure target molecules on cell surfaces. Given the complexity of molecular imprinting of large biomolecules like proteins or oligosaccharides, they opted to employ a simpler monosaccharide, glucuronic acid, as the imprinting template. Glucuronic acid is abundant on the surface of cells such as keratinocytes in the form of hyaluronan as part of the glycocalyx. The obtained fluorescently labeled glucuronic acid-imprinted NPs allowed fluorescence imaging of hyaluronan on human keratinocytes and on adult skin specimens by epifluorescence and confocal fluorescence microscopy.

### 4.6. Controlled Drug Release

Another significant use of MIPs is in the context of controlled drug delivery. The imprinting cavities of MIPs allow for the loading of drugs into the materials. In addition, their stimuli responsivity allows for fine control over the drug release rate. That is, MIPs can act as a drug reservoir for controlled release. To realize such a function, several aspects of MIPs should be taken into account. First, the structure of the imprinted cavities should be stable enough to maintain the conformation in the absence of the template, but somehow flexible enough to facilitate the attainment of a fast equilibrium between the release and re-uptake of the template in the cavity. Second, MIPs for drug delivery should be stable enough to resist enzymatic and chemical attack and mechanical stress, as the MIPs will enter into contact with biological fluids of complex compositions and different pH levels, in which the enzymatic activity is intense. Furthermore, it must be noted that the versatility of molecular imprinting technology in drug delivery also necessitates the evaluation of safety and toxicological implications.

Various applications of MIPs require specific characteristics from the produced polymers, including properties relating to morphology, specificity, capacity, binding, and release. In order to meet these requirements, various production methods for MIPs have been developed. Generally, MIPs in the form of nanospheres can be used well as carriers for drug delivery. The results of release experiments with MIP nanospheres have demonstrated highly controlled and satisfyingly paced drug release activity. Griffete and co-workers [201] developed molecularly imprinted magnetic polymer nanoparticles for the controlled release of doxorubicin (DOX) under an alternative magnetic field (AMF) in athermal conditions. Upon AMF exposure, the hydrogen bonds between the MIP and the doxorubicin were broken and the molecule was released without any significant heating of the medium. This strategy was efficient for in vitro experiments and in vivo cells. In order to coordinate the release of lysozyme, Wang and co-workers [202] prepared an NIR-light responsive lysozyme-imprinted polydopamine (PDA) layer on a fibrous SiO_2_ microsphere grown from a magnetic Fe_3_O_4_ core using oriented surface imprinting. Owing to the efficient NIR light photothermal effect of the PDA layer, the MIP-lysozyme microspheres exhibited controlled release, triggered by the NIR laser. The released lysozyme molecules maintained good bioactivity, which could efficiently decompose *E. coli*. It is important to note that sustained release is meaningless without sufficient drug release per unit time, as this could result in decreased antibiotic effectiveness or even contribute to bacterial antibiotic resistance. In order to better control release of drug, Han and co-workers recently applied dual-responsive molecularly imprinted polymers based on UiO-66-DOX to control the sustained release of DOX [203].

### 4.7. Catalysis

Because ‘from-key-to-lock’ technology generates MIPs capable of highly specific molecular recognition and catalysis, MIPs can be used as enzyme mimics. For the preparation of catalytic MIPs, the templates usually used are those of the transition state or its analogue (transition state analogue, TSA), which can generate active sites for the activation of substrate [204]. The catalytically active groups are introduced into polymers mostly by copolymerization of the appropriate monomers bearing desired catalytic functionalities (e.g., imidazole, OH, and COOH).To enhance the reduction of 4-nitrophenol(NP)to 4-aminophenol(AP), a nanoreactor with a cascade-reaction design was created using active MIP embedded with Ag or Pt nanoparticles. This MIP was produced using both NP and AP as templates. The nanoreactor catalytically converted NP into AP through hydrolysis, with the Ag or Pt nanoparticles inside then reducing the formed AP further. Unlike conventional nanoreactors focusing on one reaction, this innovative nanoreactor enables cascade reactions. The fusion of active MIPs with metal nanoparticles offers the prospect of developing catalysts suitable for complex chemical processes. Very recently, Wang and co-workers [205] proposed a novel design for a smart catalyst to be employed in the selective degradation of antibiotics and with the capability of self-reporting the degradation process by combining molecular imprinting and afterglow catalysis.

## 5. Current Challenges and Future Perspectives

Although remarkable achievements have been made in developing the synthesis methods and applications of MIPs, there still exist substantial development challenges and perspectives that need to be addressed. Firstly, the use of organic solvents in the preparation of MIPs, which are typically employed in aqueous matrices, may present a drawback. For example, one challenge that may arise during epitope imprinting is the solubility of certain peptides, particularly hydrophobic peptides prone to self-aggregation. Urraca and co-workers reported low solubility of b-amyloid peptides in different solvents and their precipitation in water. Transforming the peptide into its tetrabutylammonium salt significantly increased its solubility and imprinting could be performed [206]. Another strategy for addressing solubility issues involves the multi-epitope approach, which utilizes peptides with diverse properties [207]. Secondly, the enhancement of MIP specificity through the incorporation of other elements such as aptamers or antibodies into the recognition sites should be actively pursued. Such a hybrid approach could enhance MIP selectivity. Thirdly, the issue of reproducibility in preparation scale-up represents a barrier to commercialization. In this respect, the application of chemometric tools of experimental design for preparation optimization can be of great help. Fourthly, MIPs for macromolecules such as proteins are usually not designed with the same general principles as small molecule-based MIPs, as a protein’s conformation would be noticeably changed before the polymerization process by many frequently utilized monomers. Using a variety of materials, including hydrogels, films, monoliths, and NPs, efficient imprinting approaches have been used to produce protein-imprinting polymers. However, these protein-imprinted polymers still have a lot of issues involving compatibility and heterogeneous binding sites. Fifthly, one future perspective is that new monomers could be designed and synthesized in order to imprint molecules without functional groups, broadening the application field of MIPs. In addition, new polymerization methods for molecular imprinting will be exploited for higher imprinting efficiency and binding capacity.

## 6. Conclusions

In the last ten years, molecular imprinting technology has undergone rapid development. In the past ten years, a number of versatile and facile imprinting approaches have been invented, which have effectively solved bottleneck issues in molecular imprinting and thereby allowed for the facile production of MIPs with highly desirable properties that were not well demonstrated before. These breakthroughs have not only greatly enriched the toolbox of molecular imprinting technology, but have also significantly developed and changed the understanding and knowledge of molecular imprinting. On the other hand, this new development era has featured the tremendous incorporation of various material forms in molecular imprinting. Due to the introduction of advanced materials, such as mesoporous materials and nanoparticles, into imprinting processes, MIPs have been endowed with additional favorable properties. Because of the progress associated with the above two aspects, molecularly imprinted materials have found many promising applications. In several application areas, such as proteomics, disease diagnosis, and bio-imaging, molecularly imprinted materials have begun to show their superiority to antibodies.
